# Regulating neuronal excitability: The role of *S*-palmitoylation in Na_V_1.7 activity and voltage sensitivity

**DOI:** 10.1093/pnasnexus/pgae222

**Published:** 2024-06-04

**Authors:** Cheng Tang, Paz Duran, Aida Calderon-Rivera, Santiago Loya-Lopez, Kimberly Gomez, Samantha Perez-Miller, Rajesh Khanna

**Affiliations:** Department of Molecular Pathobiology, College of Dentistry, New York University, New York, NY 10010, USA; The National and Local Joint Engineering Laboratory of Animal Peptide Drug Development, College of Life Sciences, Hunan Normal University, Changsha 410081, China; Peptide and Small Molecule Drug R&D Platform, Furong Laboratory, Hunan Normal University, Changsha 410081, China; Department of Molecular Pathobiology, College of Dentistry, New York University, New York, NY 10010, USA; Department of Molecular Pathobiology, College of Dentistry, New York University, New York, NY 10010, USA; Department of Pharmacology and Therapeutics, College of Medicine, University of Florida, Gainesville, FL 32610, USA; Department of Molecular Pathobiology, College of Dentistry, New York University, New York, NY 10010, USA; Department of Pharmacology and Therapeutics, College of Medicine, University of Florida, Gainesville, FL 32610, USA; Department of Molecular Pathobiology, College of Dentistry, New York University, New York, NY 10010, USA; Department of Pharmacology and Therapeutics, College of Medicine, University of Florida, Gainesville, FL 32610, USA; Department of Molecular Pathobiology, College of Dentistry, New York University, New York, NY 10010, USA; Department of Pharmacology and Therapeutics, College of Medicine, University of Florida, Gainesville, FL 32610, USA; Department of Pharmacology and Therapeutics, College of Medicine, University of Florida, Gainesville, FL 32610, USA

**Keywords:** Na_V_1.7, palmitoylation, excitability, pain, human DRGs

## Abstract

*S*-palmitoylation, a reversible lipid post-translational modification, regulates the functions of numerous proteins. Voltage-gated sodium channels (Na_V_s), pivotal in action potential generation and propagation within cardiac cells and sensory neurons, can be directly or indirectly modulated by *S*-palmitoylation, impacting channel trafficking and function. However, the role of *S*-palmitoylation in modulating Na_V_1.7, a significant contributor to pain pathophysiology, has remained unexplored. Here, we addressed this knowledge gap by investigating if *S*-palmitoylation influences Na_V_1.7 channel function. Acyl-biotin exchange assays demonstrated that heterologously expressed Na_V_1.7 channels are modified by *S*-palmitoylation. Blocking *S*-palmitoylation with 2-bromopalmitate resulted in reduced Na_V_1.7 current density and hyperpolarized steady-state inactivation. We identified two *S*-palmitoylation sites within Na_V_1.7, both located in the second intracellular loop, which regulated different properties of the channel. Specifically, *S*-palmitoylation of cysteine 1126 enhanced Na_V_1.7 current density, while *S*-palmitoylation of cysteine 1152 modulated voltage-dependent inactivation. Blocking *S*-palmitoylation altered excitability of rat dorsal root ganglion neurons. Lastly, in human sensory neurons, Na_V_1.7 undergoes *S*-palmitoylation, and the attenuation of this post-translational modification results in alterations in the voltage-dependence of activation, leading to decreased neuronal excitability. Our data show, for the first time, that *S*-palmitoylation affects Na_V_1.7 channels, exerting regulatory control over their activity and, consequently, impacting rodent and human sensory neuron excitability. These findings provide a foundation for future pharmacological studies, potentially uncovering novel therapeutic avenues in the modulation of *S*-palmitoylation for Na_V_1.7 channels.

**Significance Statement**

Transmembrane Na_V_1.7 voltage-gated sodium channels contribute to the generation of action potential and are thus critical for electrical signaling of excitable cells. Post-translational modifications shape the functional properties of proteins, including Na_V_1.7. We identified that Na_V_1.7 is subject to *S*-palmitoylation, a lipid modification wherein palmitic acid is added to cysteine residues. Removal of Na_V_1.7 *S*-palmitoylation sites reduced sodium influx and changed the biophysical properties of the channels. *S*-palmitoylation also affected excitability of both rodent and human sensory neurons. While targeting protein *S*-palmitoylation holds therapeutic potential for conditions like cancer, our findings highlight its significance in Na_V_1.7, a key player in pain initiation and persistence.

## Introduction

Protein *S*-palmitoylation is a lipid modification that consists of the addition of palmitic acid (PA), a 16-carbon chain, to cysteine residues via a thioester bond (1, 2). This reversible modification is catalyzed by a family of enzymes known as palmitoyl acyltransferases (zDHHC-PATs), while their pharmacological inhibition via 2-bromopalmitate (2-BP) reduces *S*-palmitoylation. Although no consensus sequence is required for this mechanism, *S*-palmitoylation typically occurs at cysteine residues located within intracellular domains (3). *S*-palmitoylation facilitates membrane association, stability, and localization by increasing the hydrophobicity of proteins (1, 2). Among its substrates, *S*-palmitoylation targets a broad array of proteins, including voltage-gated ion channels. *S*-palmitoylation has been reported to modulate the trafficking, function, and electrophysiological characteristics of voltage-gated ion channels (4).

Voltage-gated sodium channels (VGSCs), often referred to as Na_V_s, stand out as one of the ion channel types that can be directly or indirectly influenced by this post-translational modification (4). These channels are heteromultimeric protein complexes expressed in the cell membrane responsible for regulating sodium ion influx in response to membrane depolarization (5). Mammalian Na_V_s are comprised of a large pore-forming α subunit, which typically associates with one or two auxiliary β subunits. The α subunit is composed of four homologous domains (DI–DIV), each consisting of six transmembrane helical segments (S1–S6). Within each domain, segments S1–S4 function as voltage sensors, while segments S5 and S6 form the conducting pore. These regions exhibit high sequence homology among subtypes of mammalian Na_V_s. However, the less conserved intracellular loops connecting the four homologous domains confer specificity to the nine Na_V_ isoforms (6). Na_V_s are responsible for the initiation and propagation of action potentials in excitable cells, such as neurons (5). Hence, Na_V_s play a central role in the health and disease of the nervous system.

The Na_V_1.7 isoform modulates electrogenesis in sensory neurons by determining the threshold for action potential (AP) firing. Consequently, Na_V_1.7 emerges as a central protagonist in pain signal generation and transmission (7). Notably, gain-of-function mutations within *Scn9a*, the gene encoding Na_V_1.7, underlie distinct human pain syndromes such as inherited erythromelalgia and paroxysmal extreme pain disorder (8). Moreover, chronic pain conditions often stem from the overexpression and overactivity of Na_V_1.7, making it a focal point for pain pathophysiology research. Given the limited understanding of Na_V_1.7 dysregulation, we embarked on an investigation to unveil whether *S*-palmitoylation regulates Na_V_1.7 activity. In this study, we use electrophysiological and biochemical assays to demonstrate that *S*-palmitoylation functionally regulates Na_V_1.7 in rat and human dorsal root ganglion (DRG) neurons and in a heterologous expression system. We identify two *S*-palmitoylation sites within Na_V_1.7, located in the second intracellular loop (loop 2), which regulate distinct properties of the channel. Our data indicate that *S*-palmitoylation of cysteine 1126 enhances Na_V_1.7 current density, while *S*-palmitoylation of cysteine 1152 influences the voltage dependence of inactivation. Furthermore, our study shows that blocking *S*-palmitoylation alters rat and human DRG (hDRG) neuron excitability. Therefore, our results unveil *S*-palmitoylation as a mechanism by which Na_V_1.7 activity is finely regulated.

## Results

### Regulation of sodium currents by *S*-palmitoylation in rat DRG neurons

**Fig. 1.**
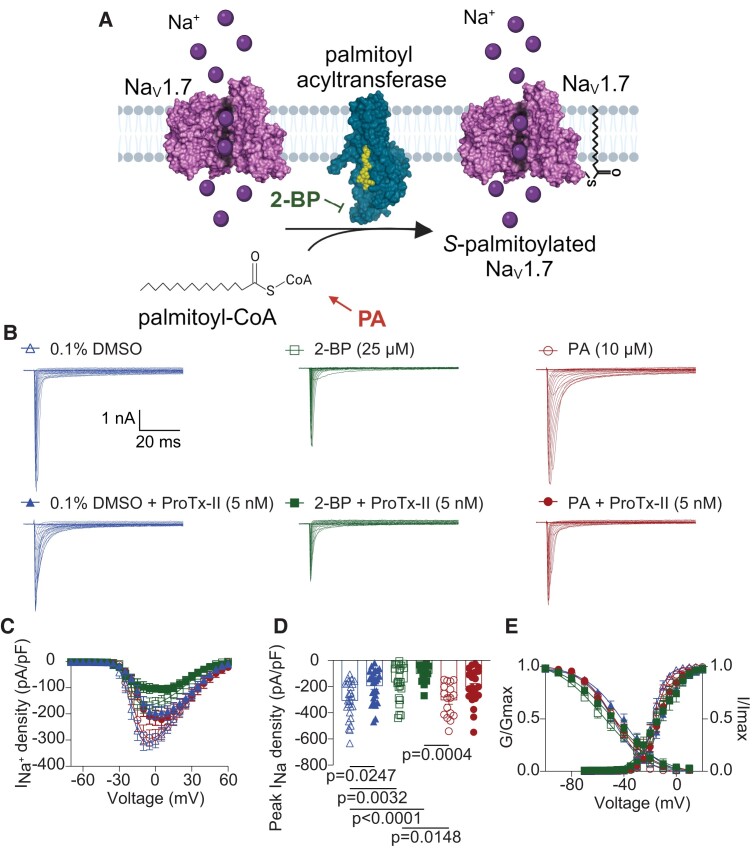
Palmitic acid (PA) and 2-Br-palmitate (2-BP) treatments alter sodium current amplitude and voltage dependence of inactivation in rat dorsal root ganglion sensory neurons. A) Illustrated schematic depicting the covalent attachment of palmitic acid (PA) to Na_V_1.7 channel via the thiolate side chain of cysteine residues. This enzymatic reaction is catalyzed by palmitoyl acyltransferases, utilizing palmitoyl-CoA as the substrate to form a reversible thioester bond. The inhibitory action of 2-bromopalmitate (2-BP) is illustrated as preventing the incorporation of palmitate onto the Na_V_1.7 channel. B) Representative traces of sodium currents (*I*_Na+_) from rat DRG neurons treated overnight with 10 μM PA (circles) to enhance *S*-palmitoylation or 25 μM 2-BP (squares) to block *S*-palmitoylation and 5 nM ProTx-II to selectively inhibit Na_V_1.7 channels. C) Summary of total I_Na+_ density versus voltage relationship. D) Bar graphs of peak *I*_Na+_ density for the indicated conditions. Boltzmann fits for normalized conductance–voltage relationship for voltage dependent activation (*G*/*G*_max_) and inactivation (*I*/*I*_max_) (E) of rat DRG neurons treated as indicated. One-way ANOVA followed by a Holm–Sidak's multiple comparisons test, *n* = 17 to 25 cells per condition from four different animals. Half-maximal potential of activation and inactivation (*V*_1/2_) and slope factor values (*k*) for activation and inactivation are presented in Table S1. Data are presented as mean ± SEM. For full statistical analyses, see Dataset S1.

To determine if Na_V_1.7 channels are regulated by *S*-palmitoylation, we performed whole-cell voltage-clamp recordings of small-to-medium diameter rat DRG neurons employing two pharmacological approaches: (i) to enhance protein *S*-palmitoylation, we increased the availability of palmitate (PA), the primary substrate for palmitoylation and (ii) 2-BP, a nonmetabolizable palmitate analog, to inhibit palmitoyltransferase activity (Fig. 1A). The 200-ms depolarization steps from −70 to +60 mV in 5 mV increments, from a holding potential of −60 mV, elicited a prototypical family of Na^+^ currents in rat DRGs (Fig. 1B). The whole-cell Na^+^ currents (pA) were normalized to cellular capacitance (pF) to obtain Na^+^ current densities (pA/pF) (Fig. 1C). We next obtained the peak current density and found that blocking *S*-palmitoylation with 25 µM of 2-BP significantly reduced total Na^+^ current density when compared to neurons treated with 0.1% DMSO as a control (Fig. 1D; DMSO: −308.3 ± 29.1 pA/pF; 2-BP: −167.8 ± 29.1 pA/pF). Interestingly, we observed that peak Na^+^ current densities in the PA-treated group did not differ from those of the control group (PA: −304.0 ± 30.4 pA/pF; Fig. 1B–D). Normalized conductance values were fitted with the Boltzmann equation to determine the voltage dependence of activation. No significant effect in conductance–voltage relationship was observed for any of the treatments (Fig. 1E and Table S1). We also assessed the channels’ steady-state fast inactivation. Figure 1E shows that blocking *S*-palmitoylation with 2-BP causes an ∼7-mV hyperpolarizing shift of steady-state inactivation (DMSO: *V*_1/2_ = −48.5 ± 1.0 mV; PA: *V*_1/2_ = −47.0 ± 1.0 mV; 2-BP: *V*_1/2_ = −55.4 ± 2.0 mV; Table S1). In addition, 2-BP reduced the slope of the voltage dependence of fast inactivation (2-BP: −18.86 ± 2.28 vs. DMSO −11.28 ± 0.89).

We next used Protoxin-II (ProTx-II), a selective Na_V_1.7 channel blocker, to determine the contribution of Na_V_1.7 channels to the reduction in total Na^+^ currents induced by 2-BP treatment. Acute application of ProTx-II reduced Na^+^ currents and peak current density both in the control (DMSO + ProTx-II: −195.8 ± 24.05 pA/pF) and PA-treated (PA + ProTX-II: −203.4 ± 27.9 pA/pF) groups (Fig. 1B–D). These findings indicated that the current density attributed to ProTx-II-sensitive Na_V_1.7 channels in these two groups was approximately 100–110 pA/pF (Fig. 1D and Table S1). While ProTx-II also reduced total Na^+^ current density of 2-BP-treated channels (2-BP + ProTx-II: 103.0 ± 18.8 pA/pF), the current densities of 2-BP and 2-BP + ProTx-II treated groups were no longer significantly different (Fig. 1B–D). Furthermore, the calculated current density contributed by Na_V_1.7 channels decreased to approximately 64 pA/pF following 2-BP treatment (Fig. 1D and Table S1). These data strongly suggested that the inhibition in the palmitoylation by 2-BP silenced a substantial portion of Na_V_1.7 channels. In summary, these data show that *S*-palmitoylation regulates Na_V_1.7 functional properties in rodent DRG sensory neurons.

These findings prompted us to investigate the effect of *S*-palmitoylation of Na_V_1.7 channels in a more controlled setting, as other VGSCs (4, 9) and auxiliary subunits (10, 11) present in sensory neurons are palmitoylated and could potentially be responsible for these observations. Therefore, we proceeded to conduct electrophysiological experiments in a heterologous expression system.

### *S*-palmitoylation modulates Na_V_1.7 current amplitude and voltage dependence of inactivation in HEK293 cells

**Fig. 2.**
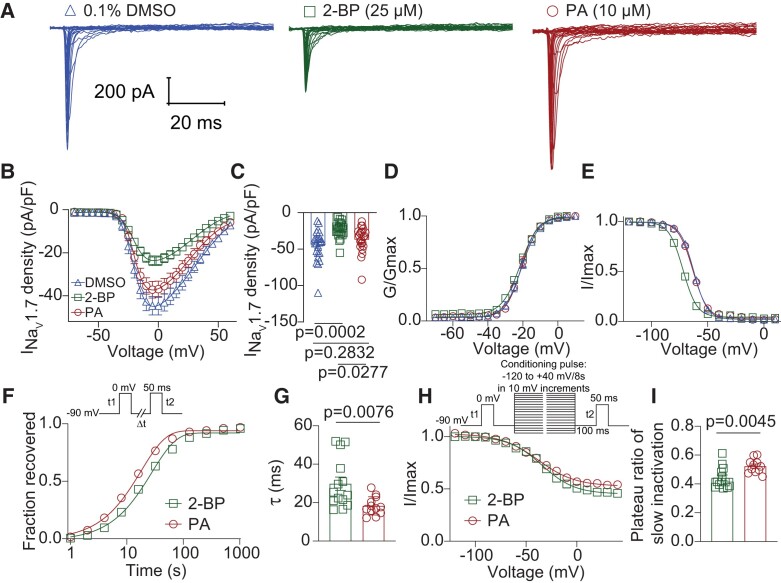
Post-translational *S*-palmitoylation regulates Na_V_1.7 current amplitude and biophysical properties in HEK293 cells. A) Representative traces of Na_V_1.7 currents from HEK293 cells expressing Na_V_1.7 channels and treated overnight with 0.1% DMSO (triangles) as control, 10 μM PA (circles) to enhance *S*-palmitoylation, or 25 μM 2-BP (squares) to block *S*-palmitoylation. B) Summary of Na_V_1.7 density vs. voltage relationship (*V_h_* = −110 mV). (C) Bar graphs of peak Na_V_1.7 current density for the indicated conditions showing that 2-BP significantly decreased Na_V_1.7 currents when compared to the control (one-way ANOVA followed by a Tukey's post hoc test, *n* = 22 to 24 cells per condition). Voltage dependence of steady-state activation (*G*/*G*_max_) (D) and fast inactivation (*I*/*I*_max_) (E) of Na_V_1.7 channels expressed in HEK293 cells and treated as indicated. 2-BP treatment caused a hyperpolarizing shift in the voltage dependence of steady-state fast inactivation. Half-maximal potential of activation and inactivation (*V*_1/2_) and slope factor values (*k*) for activation and inactivation are presented in Table S2. Time-course (F) and calculated time constant (*τ*) (G) of recovery from inactivation for Na_V_1.7 channels treated as indicated, inset in (F) shows the voltage protocol. Voltage dependence of steady-state slow inactivation (H) of Na_V_1.7 channels treated as indicated, with Boltzmann fitting of the curves determining the plateau ratio of noninactivated channels (I), plateau ratio for 2-BP (44.1 ± 2.1%) and PA (52.4 ± 1.5%) groups. Inset in (H) shows the voltage protocol. Data are presented as mean ± SEM. For full statistical analyses, see Dataset S1.

To study in more detail how *S*-palmitoylation regulates Na_V_1.7 channel function, we transiently transfected mouse Na_V_1.7 cDNA without β subunits into HEK293 cells and subsequently treated the cells with 0.1% DMSO (as control), 10 µM PA, or 25 µM 2-BP and performed whole-cell electrophysiological recordings. Figure 2A shows representative traces of Na_V_1.7 currents from all treatments. 2-BP treatment substantially decreased Na_V_1.7 current density compared to the control condition, while PA treatment had no significant effect (Fig. 2B and C; DMSO: −44.6 ± 4.3 pA/pF; PA: −37.0 ± 3.8 pA/pF; 2-BP: −23.7 ± 2.2 pA/pF). We also examined whether *S*-palmitoylation affects Na_V_1.7 voltage dependence of activation (Fig. 2D and Table S2) and inactivation (Fig. 2E and Table S2). No significant change was observed in the conductance–voltage relationship between groups (Fig. 2D and Table S2; DMSO: *V*_1/2_ = −20.6 ± 0.4 mV; PA: *V*_1/2_ = −20.9 ± 0.4 mV; 2-BP: *V*_1/2_ = −21.5 ± 0.3 mV). However, 2-BP treatment shifted the steady-state inactivation to more hyperpolarized voltages compared to the control condition (Fig. 2E and Table S2; DMSO: *V*_1/2_ = −62.5 ± 0.3 mV; PA: *V*_1/2_ = −63.2 ± 0.4 mV; 2-BP: *V*_1/2_ = −72.0 ± 0.3 mV). These results confirm that the function of heterologously expressed Na_V_1.7 channels is regulated by *S*-palmitoylation. Interestingly, our results in HEK293 cells confirm our findings observed in rat DRGs, suggesting that increasing the availability of *S*-palmitoylation with PA treatment causes no significant effect on Na_V_1.7 channel function (Fig. 2A–E). This absence of modulation could potentially be caused by the saturation of *S*-palmitoylation-mediated regulation of Na_V_1.7 in both the heterologous expression system and DRG neurons. Thus, for our subsequent experiments, we opted to compare the effects of PA with those of 2-BP treatment.

To investigate whether S-palmitoylation regulates other functional properties of Na_V_1.7 channels, we next explored if *S*-palmitoylation regulates Na_V_1.7 time-dependent recovery from fast inactivation. The fast-repriming kinetics of Na_V_1.7 channels were measured by applying the following protocol; from a holding potential of −90 mV, a first depolarizing test pulse to 0 mV for 50 ms (*t*1) was applied to inactivate all the Na_V_1.7 channels, then a conditioning pulse was applied at the recovery voltage (−90 mV) for increasing recovery durations prior to the second test pulse to 0 mV (*t*2) to measure the recovered channels. The fraction recovered was determined by normalizing the currents at *t*2 to that at *t*1 (*I_t_*_2_/*I_t_*_1_) and then plotted against the durations of recovery potential (Fig. 2F), and exponential fits were used to obtain the recovery time constants (*τ*). Figure 2G shows that recovery time constant for cells treated with 2-BP was significantly slower compared to cells treated with PA (PA: *τ* = 18.3 ± 1.4 ms; 2-BP: *τ* = 29.8 ± 3.5 ms).

Slow inactivation of VGSCs regulates excitability by reducing the number of channels available to open. To assess the steady-state slow inactivation (SSI) of Na_V_1.7 channels, we determined the availability of noninactivated channels after clamping the channels at various voltages (−120 mV to +40 mV, in 10 mV increment) for a prolonged time of 8 s. Specifically, we measured the currents before (*I_t_*_1_) and after (*I_t_*_2_) the conditioning pulse and plotted the *I_t_*_2_/*I_t_*_1_ ratio as a function of voltage. This ratio gradually decreased with increasing conditioning voltage steps tested until it eventually reached a plateau. Analysis of the SSI curve showed that, although 2-BP treatment did not affect the voltage-dependence of Na_V_1.7 slow inactivation, it did enhance the channel's slow inactivation as demonstrated by a decreased plateau ratio of available channels at positive voltages (Fig. 2H and I). Together, these results show that *S*-palmitoylation functionally regulates Na_V_1.7 current amplitude, voltage-dependence of fast inactivation, fast-repriming kinetics, as well as slow inactivation in the HEK293 heterologous expression system.

### Na_V_1.7 is post-translationally modified by *S*-palmitoylation

**Fig. 3.**
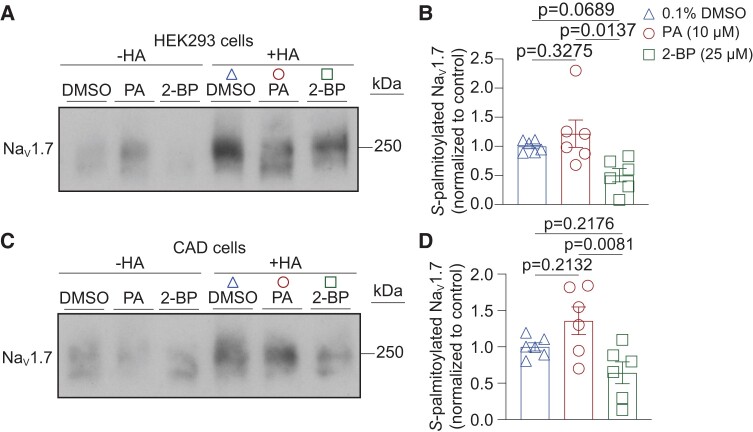
Palmitate lipid treatment alters Na_V_1.7 palmitoylation in HEK293 and CAD cells. The acyl-biotin exchange (ABE) assay was used to detect protein palmitoylation. Representative immunoblots of ABE assay on HEK293 cells expressing Na_V_1.7 channels (A) and mouse catecholamine A differentiated (CAD) cells (C) treated for 24 h with 0.1% DMSO (triangles) as control, 10 μM PA (circles) to enhance *S*-palmitoylation, or 25 μM 2-BP (squares) to block *S*-palmitoylation. Na_V_1.7 palmitoylation was identified where +HA (hydroxylamine treated group) indicates the existence of protein palmitoylation on Na_V_1.7 channels and −HA (tris treated group) serves as the negative experimental control. A Na_V_1.7 antibody was used to detect biotinylated (previously palmitoylated) and precipitated channels using streptavidin pull down assay. Bar graph with scatter plots showing the quantification of the ABE assay on HEK293 cells expressing Na_V_1.7 channels (B) and CAD (D) cells and treated as indicated. One-way ANOVA followed by a Tukey's post hoc test, *n* = 6 samples. *P* values as indicated; Data are presented as mean ± SEM. For full statistical analyses, see Dataset S1.

Our results so far suggest that manipulating *S*-palmitoylation with pharmacological treatments alters Na_V_1.7 channel function. However, it is unknown whether Na_V_1.7 channels are directly modified by *S*-palmitoylation. To investigate this, we used the acyl-biotin exchange (ABE) assay (12), an in vitro technique that detects thioester-linked acyl-modifications, such as *S*-palmitoylation, by using hydroxylamine to cleave the thioester bond and substituting it with a biotinyl moiety. We tested proteins extracted from HEK293 cells heterologously expressing Na_V_1.7 channels and mouse catecholamine A differentiated (CAD) cells with endogenous Na_V_1.7 expression (13) and found that Na_V_1.7 channels are indeed *S*-palmitoylated (Fig. 3A and C). The signal in the hydroxylamine treated protein extracts (“+HA” lane) represent the *S*-palmitoylated proteins before hydroxylamine thioester-cleavage. As a negative control for HA treatment, an equal portion of the protein extract was processed without hydroxylamine treatment to rule out false-positive identifications of palmitoylated proteins. The absence of a signal in the “−HA” lane indicates that the signal in “+HA” lane is dependent of hydroxylamine activity and reflects frank *S*-palmitoylation. A 24 h treatment of the cells with PA (10 µM) slightly enhanced Na_V_1.7 *S*-palmitoylation (normalized to DMSO, +HA treatment), while treatment with 2-BP (25 µM) to block *S*-palmitoylation reduced Na_V_1.7 *S*-palmitoylation (Fig. 3A–D). These findings imply that both heterologously expressed and native Na_V_1.7 channels undergo S-palmitoylation.

### *S*-palmitoylation of Na_V_1.7 regulates current amplitude (at Cys1126) and voltage dependence of inactivation (at Cys1152) in HEK293 cells

**Fig. 4.**
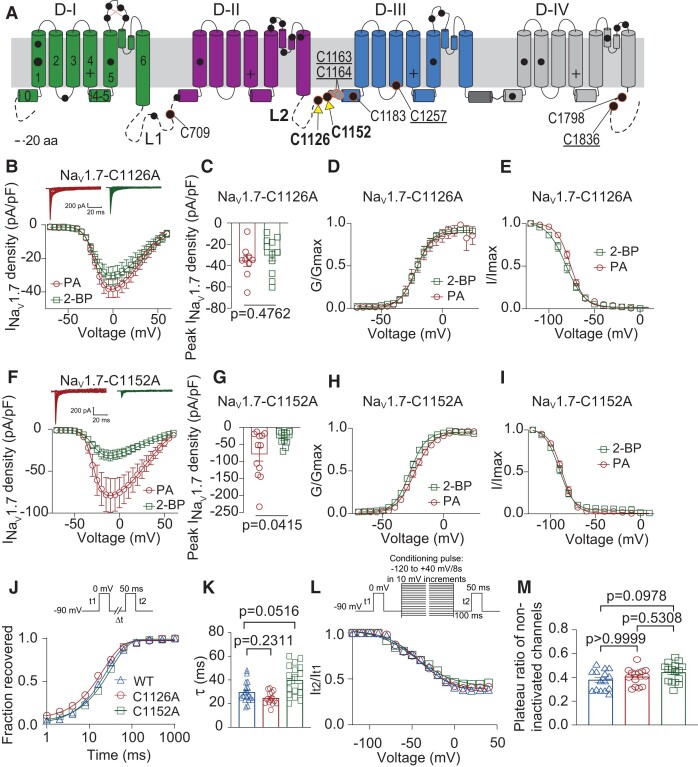
*S*-palmitoylation of Cys1126 and Cys1152 regulates Na_V_1.7 current amplitude and voltage dependence of inactivation. A) Domain diagram of Na_V_1.7 showing locations of all cysteines in the mouse isoform as filled black circles. Orange outlined the sites tested with alanine mutations. Underlined labels indicate sites predicted to be palmitoylated by CSS-PALM-4.0 (14) and with intracellular localization. A full list of predicted sites can be found in the methods. B) Representative traces and summary current density–voltage relationship of Na_V_1.7-C1126A channels expressed in HEK293 cells and treated overnight with 10 μM PA (circles), or 25 μM 2-BP (squares), *V_h_* = −110 mV. C) Bar graphs of peak Na_V_1.7-C1126A current density for the indicated conditions (unpaired *t* test, *n* = 9 to 11 cells per condition). Voltage dependence of steady-state activation (*G*/*G*_max_) (D) and inactivation (*I*/*I*_max_) (E) of Na_V_1.7-C1126A channels expressed in HEK293 cells and treated as indicated. F) Representative traces and summary current density–voltage relationship of Na_V_1.7-C1152A channels expressed in HEK293 cells and treated overnight with 10 μM PA (circles), or 25 μM 2-BP (squares). G) Bar graphs of peak Na_V_1.7-C1152A current density for the indicated conditions (unpaired *t* test, n = 11 to 12 cells per condition). Voltage dependence of steady-state activation (*G*/*G*_max_) (H) and inactivation (*I*/*I*_max_) (I) of Na_V_1.7-C1152A channels expressed in HEK293 cells and treated as indicated. Time-course (J) and calculated time constant (*τ*) (K) of recovery from inactivation for Na_V_1.7 WT channels as well as Na_V_1.7-C1126A and Na_V_1.7-C1152A mutants, inset in (J) shows the voltage protocol. Voltage dependence of steady-state slow inactivation (L) of Na_V_1.7 WT channels as well as Na_V_1.7-C1126A and Na_V_1.7-C1152A mutants, with Boltzmann fitting of the curves determining the plateau ratio of noninactivated channels (M), inset in (L) shows the voltage protocol. Half-maximal potential of activation and inactivation (*V*_1/2_) and slope factor values (*k*) for activation and inactivation are presented in Table S2. Data are presented as mean ± SEM. For full statistical analyses, see Dataset S1.

To search for potential *S*-palmitoylation sites in mouse Na_V_1.7, we next used CSS-Palm-4.0, a palmitoylation site prediction software (14). The entire Na_V_1.7 sequence was analyzed, and nine cysteine sites were identified at medium threshold. Four of these sites (C1163, C1164, C1257, and C1836) are accessible from the intracellular side of the plasma membrane (Fig. 4A). Residues C1163 and C1164 correspond to the major palmitoylation sites C1169/C1170 identified for mouse Na_V_1.6 (9) and two (C1178/C1179) of the four sites predicted for human Na_V_1.5 (15) (Fig. 4A). To test whether these cysteines in Na_V_1.7 have a functional role in regulating channel *S*-palmitoylation, we used site-directed mutagenesis to construct mutant channels with cysteine residues changed to nonpalmitoylatable alanine residues. We transiently transfected the mutant channel constructs into HEK293 cells and subsequently treated them with 10 µM PA or 25 µM 2-BP.

Electrophysiological recordings indicated that 2-BP treatment decreased peak Na_V_1.7 current density and shifted voltage-dependence of inactivation for these four mutant constructs (Fig. S1; C1163A, C1164A, C1257A, and C1836A), similar to the effects observed for Na_V_1.7-WT channels. Since mutation of the predicted cysteines to alanine showed that none were the sites of *S*-palmitoylation in Na_V_1.7, we next identified all additional cysteines that could be potentially accessible to the palmitoylation machinery (C177, C623, C709, C725, C1126, C1152, C1157, C1183, C1505, and C1798) and tested five with alanine mutations (C709A, C1126A, C1152A, C1183A, and C1798A) (Fig. 4A). We found that 2-BP treatment also significantly reduced the peak current density and shifted the voltage-dependent inactivation of the C709A, C1183A, and C1798A mutant channels (Fig. S1).

However, electrophysiological recordings of HEK293 cells transfected with Na_V_1.7-C1126A mutant channel showed that regulation of Na_V_1.7 current density by 2-BP treatment was lost by the mutation of this cysteine (Fig. 4B and C; PA: −35.74 ± 5.12 pA/pF; 2-BP: −30.36 ± 5.21 pA/pF). Similar to the Na_V_1.7-WT channels, no change on voltage-dependence of activation was observed between treatments (Fig. 4D and Table S2). Interestingly, compared to Na_V_1.7-WT, 2-BP treatment resulted in a smaller but still significant hyperpolarizing shift of the steady-state inactivation in the Na_V_1.7-C1126A mutant channel (Fig. 4E and Table S2; PA: *V*_1/2_ = −76.30 ± 0.57 mV; 2-BP: *V*_1/2_ = −81.45 ± 0.95 mV), coupled with a remarkable reduction of the slope factor (PA: *k* = −7.7 ± 0.49 mV; 2-BP: *k* = −10.29 ± 0.81 mV). These findings implied that S-palmitoylation of cysteine 1126 played a partial, though not central role in regulating the voltage-dependence of inactivation of Na_V_1.7. We observed that Na_V_1.7-C1152A displayed the same current density modulation produced by 2-BP treatment similar to Na_V_1.7-WT (Fig. 4F and G; PA: −78.99 ± 20.04 pA/pF; 2-BP: −31.67 ± 6.21 pA/pF). No change was observed in the conductance–voltage relationship (Fig. 4H and Table S2). Unexpectedly, regulation of the steady-state inactivation by 2-BP was lost in Na_V_1.7-C1152A channels (Fig. 4I and Table S2; PA: *V*_1/2_ = −87.62 ± 0.54 mV; 2-BP: *V*_1/2_ = −90.47 ± 0.86 mV). As inhibiting S-palmitoylation with 2-BP also altered Na_V_1.7's repriming kinetics and steady-state inactivation (Fig. 2F–I), we sought to explore whether the C1126A and C1152A mutations could abolish this effect. Surprisingly, our findings indicate that these two mutations did not elicit any noticeable alterations in either the recovery from inactivation or the SSI of Na_V_1.7 channels (Fig. 4J–M). Together, our results show that *S*-palmitoylation at different cysteines distinctively regulates different biophysical properties of Na_V_1.7.

**Fig. 5.**
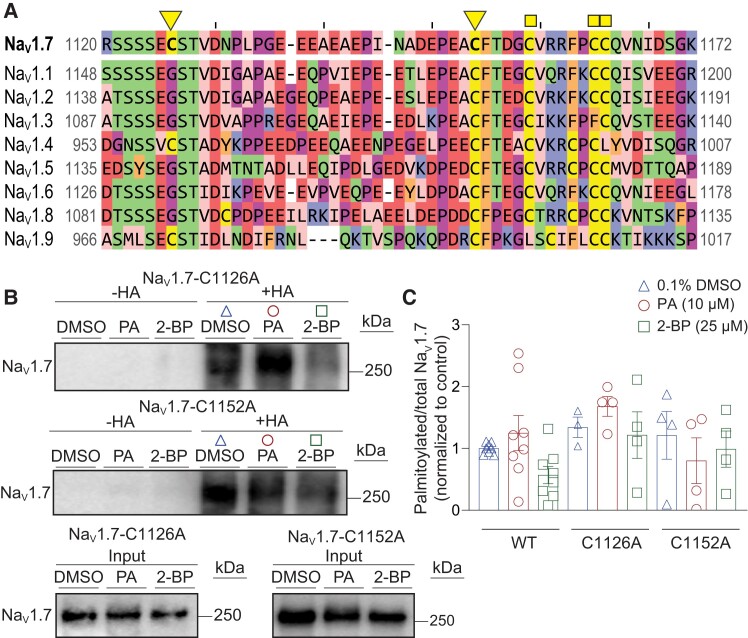
Characterization of Na_V_1.7-C1126A and Na_V_1.7-C1152A palmitoylation using ABE method. A) Sequence alignment of mouse Na_V_ isoforms in the region of newly identified and known palmitoylation sites. Inverted triangles mark the Na_V_1.7 palmitoylation sites identified in this paper and squares mark previously published Na_V_1.5 (15) and Na_V_1.6 (9) palmitoylation sites. The region shown lies at the C-terminal end of intracellular Loop 2. B) Representative immunoblots of ABE assay on HEK293 cells expressing Na_V_1.7-C1126A or Na_V_1.7-C1152A channels and treated for 24 h with 0.1% DMSO (triangles) as control, 10 μM PA (circles) to enhance *S*-palmitoylation, or 25 μM 2-BP (squares) to block *S*-palmitoylation. Na_V_1.7 palmitoylation was identified where +HA (hydroxylamine treated group) indicates the existence of protein palmitoylation on Na_V_1.7 channels and −HA (tris treated group) serves as the negative experimental control. C) Bar graph with scatter plots showing the quantification of the ABE assay on HEK293 cells expressing Na_V_1.7-C1126A or Na_V_1.7-C1152A channels and treated as indicated. Data are presented as mean ± SEM. For full statistical analyses, see Dataset S1.

Sequence alignment of the C-terminal region of loop 2 for Na_V_ isoforms (Fig. 5A), highlights both the newly identified and previously known *S*-palmitoylation cysteine residues (9, 15). Among these residues, Cys1152 is found to be highly conserved across all nine Na_V_ isoforms. However, Cys1126 is only observed in Na_V_1.4, Na_V_1.9, and Na_V_1.7 isoforms, suggesting an isoform-specific *S*-palmitoylation regulation of Na_V_ channels. To biochemically confirm that *S*-palmitoylation occurs at Cys1126 and Cys1152, we assessed the *S*-palmitoylation signal of Na_V_1.7-C1126A and Na_V_1.7-C1152A channels using the heterologous expression system and the ABE assay. Figure 5B demonstrates that substituting either of the cysteine residues with alanine had no discernible impact on the Na_V_1.7 *S*-palmitoylation signal. Quantification of the Na_V_1.7 signal in the hydroxylamine treated protein extracts (“+HA” lane) indicated that *S*-palmitoylation of Na_V_1.7-C1126A was slightly increased by PA treatment but not affected by 2-BP treatment (Fig. 5C). Whereas *S*-palmitoylation of Na_V_1.7-C1152A was reduced by both PA and 2-BP treatments (Fig. 5C). These seemingly conflicting results might be attributed to the presence of the other *S*-palmitoylation site within these single mutant constructs. Moreover, these findings are in line with the electrophysiological data demonstrating that both the C1126 and C1152 residues in Na_V_1.7 were palmitoylated.

### Loss of *S*-palmitoylation of Na_V_1.7 at Cys1126 and Cys1152 reduces channel activity in HEK293 cells

Our data provides evidence that *S*-palmitoylation of Cys1126 and Cys1152 distinctly regulates the functional expression and kinetics of Na_V_1.7 channels. However, when we substituted these cysteine residues with alanine, we observed that the *S*-palmitoylation signal was not eliminated in the ABE assays (Fig. 5B and C). Hence, we postulated that S-palmitoylation of both cysteine residues, 1126 and 1152, plays a crucial role in governing specific aspects of Na_V_1.7 function. Consequently, mutating both sites simultanesously could potentially completely counteract the functional effects observed during PA and 2-BP treatments. To test this hypothesis, we substituted these two cysteine residues with alanine to eliminate endogenous *S*-palmitoylation. Subsequently, we examined the functional properties of this double mutant channel construct, referred to as Na_V_1.7-C1126A/C1152A, in our heterologous expression system.

**Fig. 6.**
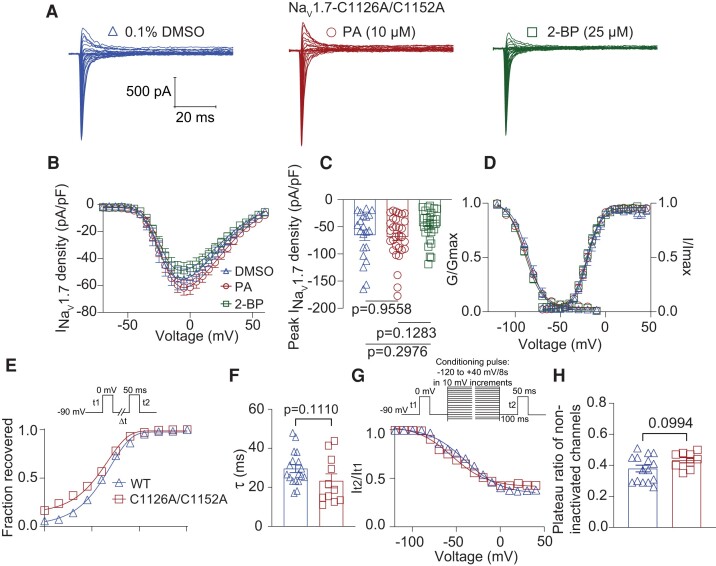
Na_V_1.7-C1126A/C1152A mutations eliminate current and voltage dependence response to S-palmitoylation manipulation. A) Representative traces of Na_V_1.7 currents from HEK293 cells expressing Na_V_1.7-C1126A/C1152A channels and treated overnight with 0.1% DMSO (triangles) as control, 10 μM PA (circles) to enhance *S*-palmitoylation, or 25 μM 2-BP (squares) to block *S*-palmitoylation. B) Summary of Na_V_1.7-C1126A/C1152A density vs. voltage relationship, *V_h_* = −110 mV. C) Bar graphs of peak Na_V_1.7-C1126A/C1152A current density for the indicated conditions (one-way ANOVA followed by a Tukey's post hoc test, *n* = 22 to 24 cells per condition). D) Voltage dependence of steady-state activation (*G*/*G*_max_) and inactivation (*I*/*I*_max_) of Na_V_1.7-C1126A/C1152A channels expressed in HEK293 cells and treated as indicated. Time-course (E) and calculated time constant (*τ*) (F) of recovery from inactivation for Na_V_1.7 WT channels as well as Na_V_1.7-C1126A/C1152A mutant, inset in (E) shows the voltage protocol. Voltage dependence of steady-state slow inactivation (G) of Na_V_1.7 WT channels as well as Na_V_1.7-C1126A/C1152A mutant, with Boltzmann fitting of the curves determining the plateau ratio of noninactivated channels (H), inset in (G) shows the voltage protocol. Half-maximal potential of activation and inactivation (*V*_1/2_) and slope factor values (*k*) for activation and inactivation are presented in Table S2. Data are presented as mean ± SEM. For full statistical analyses, see Dataset S1.

HEK293 cells were transiently transfected with Na_V_1.7-C1126A/C1152A, and treated with 0.1% DMSO (as control), 10 µM PA or 25 µM 2-BP. Whole-cell electrophysiological recordings were performed, obtaining representative traces of Na_V_1.7-C1126A/C1152A current from each treatment group (Fig. 6A). Our findings reveal that 2-BP treatment no longer decreased Na_V_1.7 current density (Fig. 6B and C; DMSO: −65.40 ± 9.79 pA/pF; PA: −68.50 ± 7.14 pA/pF; 2-BP: −48.74 ± 5.77 pA/pF). Furthermore, the modulation of the voltage-dependence of inactivation by 2-BP was eliminated in Na_V_1.7-C1126A/C1152A channels (Fig. 6D and Table S2; DMSO: *V*_1/2_ = −85.37 ± 0.98 mV; PA: *V*_1/2_ = −87.92 ± 0.95 mV; 2-BP: *V*_1/2_ = −89.45 ± 0.99 mV). Importantly, there were no significant differences in the conductance–voltage relationship between treatment groups (Fig. 6D and Table S2; DMSO: *V*_1/2_ = −20.19 ± 0.79 mV; PA: *V*_1/2_ = −20.28 ± 0.43 mV; 2-BP: *V*_1/2_ = −20.68 ± 0.66 mV). We further evaluated if fast-repriming kinetics and SSI were affected in the effect double mutants. Na_V_1.7-C1126A/C1152A showed no appreciable disparities in the recovery from inactivation compared to WT-Na_V_1.7 channels (Fig. 6E and F). However, a statistically significant difference in the plateau ratio of available channels was noted (Fig. 6G and H).

**Fig. 7.**
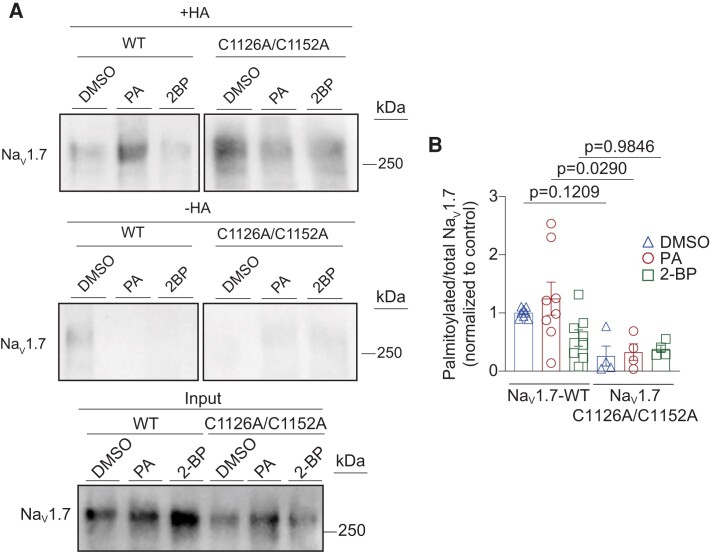
Na_V_1.7-C1126A/C1152A mutation prevents Na_V_1.7 S-palmitoylation in HEK293 cells. A) Representative immunoblots of ABE assay on HEK293 cells expressing Na_V_1.7-WT or Na_V_1.7-C1126A/C1152A channels and treated for 24 h with 0.1% DMSO (triangles) as control, 10 μM PA (circles) to enhance *S*-palmitoylation, or 25 μM 2-BP (squares) to block *S*-palmitoylation. Na_V_1.7 palmitoylation was identified where + HA (hydroxylamine treated group) indicates the existence of protein palmitoylation on Na_V_1.7 channels and −HA (tris treated group) serves as the negative experimental control. B) Bar graph with scatter plots showing the quantification of the ABE assay on HEK293 cells expressing Na_V_1.7-WT or Na_V_1.7-C1126A/C1152A channels and treated as indicated. One-way ANOVA followed by a Tukey's post hoc test, *n* = 4 to 8 samples. Data are presented as mean ± SEM. For full statistical analyses, see Dataset S1.

To biochemically confirm that the lack of effect observed with 2-BP treatment is indeed due to the elimination of palmitoylation at C1126 and C1152, we conducted ABE assays to assess the *S*-palmitoylation status of Na_V_1.7-WT and Na_V_1.7-C1126A/C1152A. For this, HEK293 cells were transiently transfected with the Na_V_1.7 constructs and subsequentially treated with 0.1% DMSO (as control), 10 µM PA or 25 µM 2-BP. Our results demonstrate that the substitution of these cysteine residues substantially reduced the *S*-palmitoylation of the channel (Fig. 7A and B). These findings strongly support the hypothesis that modification of these two endogenous cysteines residues by *S*-palmitoylation induces changes in Na_V_1.7 function.

### *S*-palmitoylation modulates excitability of rat DRG neurons

**Fig. 8.**
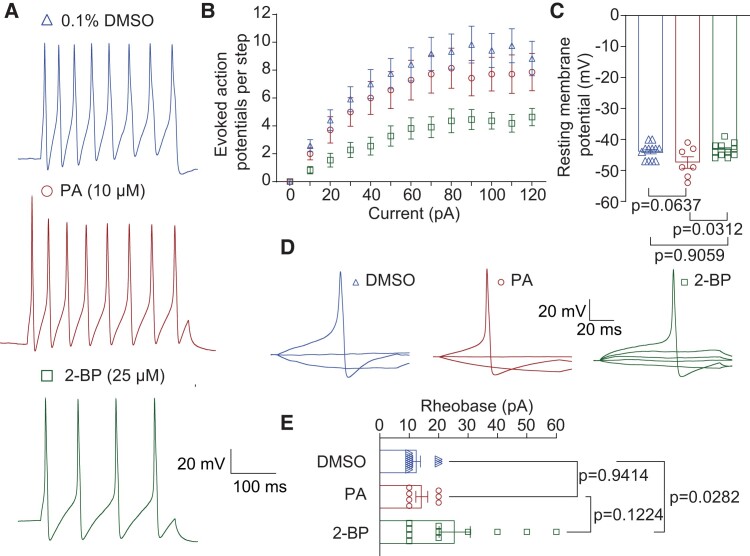
Chemical inhibition of palmitoylation decreases excitability of rat dorsal root ganglion sensory neurons. A) Representative traces of evoked action potentials at 120 pA from rat dorsal root ganglion neurons (DRGs) treated overnight with 0.1% DMSO (triangles) as control, 10 μM PA (circles) to enhance *S*-palmitoylation, or 25 μM 2-BP (squares) to block *S*-palmitoylation. B) Summary of the number of current-evoked action potentials in response to a depolarizing ramp stimulus from 0 to 120 pA of current injection of the indicated conditions. C) Quantification of the resting membrane potential in millivolts (mV) in the in the presence of overnight 0.1% DMSO, PA, or 2-BP. Representative traces (D) and quantification (E) of the rheobase in picoamperes (pA) of neurons treated as indicated. One-way ANOVA followed by a Tukey's multiple comparisons test was performed for the resting membrane potential and rheobase data, *n* = 7 to 12 cells per condition. A two-way ANOVA followed by a Tukey's multiple comparisons test was performed for the 0–120 pA-step excitability protocol, *n* = 7 to 12 cells per condition. Data are presented as mean ± SEM. For full statistical analyses, see Dataset S1.

Given Na_V_1.7's contribution to AP firing within sensory neurons, we next examined whether changes in *S*-palmitoylation of Na_V_1.7 channels might contribute to alterations in neuron excitability. First, we examined the excitability properties of rat DRG neurons following treatment with PA and 2-BP. DRG APs were elicited by applying depolarizing current injections ranging from 0 to 120 pA with an increment of 10 pA in 300 ms (Fig. 8A). Notably, a decrease in *S*-palmitoylation induced by 2-BP treatment resulted in a reduction of evoked AP frequency across all tested current injections (Fig. 8B) when compared to neurons treated with 0.1% DMSO as a control. Furthermore, the resting membrane potential was decreased by PA treatment ∼4 mV. Although this change was significantly different from neurons treated with 2-BP, it was not statistically lower than the control condition (Fig. 8C; DMSO: −43.92 ± 0.74 mV; PA: −47.43 ± 1.84 mV; 2-BP: −43.36 ± 0.66 mV). The rheobase, defined as the minimum current necessary to evoke an AP, was significantly increased in neurons treated with 2-BP. However, no significant change was observed in neurons treated with PA (Fig. 8D and E; DMSO: 12.50 ± 1.31 pA; PA: 14.29 ± 2.02 pA; 2-BP: 25.45 ± 5.29 pA). These findings collectively demonstrate that reducing *S*-palmitoylation can attenuate DRG neuron excitability.

### *S*-palmitoylation regulates human neuronal excitability via modulation of voltage-dependence of activation of sodium currents

To gain insights into the translational relevance of *S*-palmitoylation of Na_V_1.7, we conducted the ABE assay using spinal cord lysates obtained from human donors (see demographics of donors in Table S3). Positive signals, indicative of Na_V_1.7 *S*-palmitoylation, were observed in samples treated with hydroxylamine, contrasting with the absence of such signals in negative control samples (Fig. S2). These findings suggest that human Na_V_1.7 channels are *S*-palmitoylated.

**Fig. 9.**
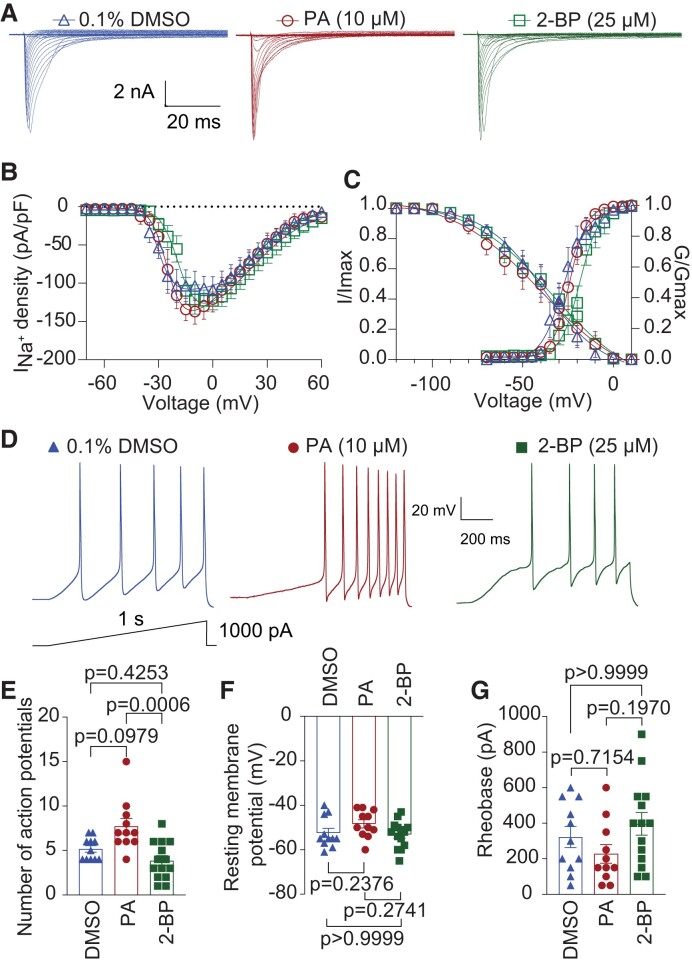
2-BP treatment alters the voltage-dependence of activation of sodium currents and decreases human DRG neuron excitability. A) Representative traces of sodium currents (*I*_Na+_) from human DRG neurons treated overnight with 0.1% DMSO (triangles), 10 μM PA (circles) or 25 μM 2-BP (squares). B) Summary of total *I*_Na_^+^ density versus voltage relationship. (C) Boltzmann fits for normalized conductance–voltage relationship for voltage dependent activation (*G*/*G*_max_) and inactivation (*I*/*I*_max_) of human DRG neurons treated as indicated. One-way ANOVA followed by a Tukey's post hoc test, *n* = 7 to 9 cells per condition from four donors, see Table S4. Half-maximal potential of activation and inactivation (*V*_1/2_) and slope factor values (*k*) for activation and inactivation are presented in Table S5. D) Sample traces of action potential firing in human DRG neurons evoked by injecting a 1 s ramp pulse from 0 to 1000 pA. E) Summary of the number of evoked action potentials in response to a depolarizing ramp stimulus from 0 to 1,000 pA. Quantification of the resting membrane potential (F) and rheobase (G) of neurons treated with 0.1% DMSO (blue triangles) as control, 10 μM PA (red circles) to enhance *S*-palmitoylation, or 25 μM 2-BP (green squares) to block *S*-palmitoylation. *N* = 11–14 cells; *P* value as indicated; Kruskal–Wallis test followed by Dunn's multiple comparison test. Data are presented as mean ± SEM. For full statistical analyses, see Dataset S1.

To elucidate if sodium channels are functionally regulated by *S*-palmitoylation in human DRG (hDRG) neurons, we acquired dissociated hDRG neurons from AnaBios Corporation (see demographics of donors in Table S4). Next, we tested whether overnight treatment with PA (10 µM) and 2-BP (25 µM) influence sodium current densities and/or voltage-dependence of activation and inactivation. Whole-cell sodium currents were elicited by 200-ms depolarization steps from −70 to +60 mV in 5 mV increments, from a holding potential of −60 mV (Fig. 9A). Current density–voltage curves (Fig. 9B) and peak current density (Table S5) demonstrate no significant differences between groups. Interestingly, when compared to DMSO- and PA-treated cells, 2-BP caused a significant depolarizing shift of ∼9 mV and ∼6 mV, respectively, of the midpoint potential for activation (DMSO: *V*_1/2_ = −27.52 ± 1.26 mV; PA: *V*_1/2_ = −24.12 ± 0.77 mV; 2-BP: *V*_1/2_ = −18.48 ± 0.92 mV; Table S5), while the voltage dependence of steady-state fast inactivation was unchanged (Fig. 9C).

To explore if this shift in the voltage-dependence of activation could attenuate human neuronal excitability, we treated hDRGs overnight with 0.1% DMSO (as control), 10 µM PA, or 25 µM 2-BP, and action potentials were evoked by a ramp pulse from 0–1000 pA in 1 sec as demonstrated in Fig. 9D. Application of PA did not lead to significant changes in the number of action potentials when compared to the control group (Fig. 9E). However, when *S*-palmitoylation was decreased using 2-BP, the number of action potentials was significantly reduced when compared to PA-treated cells (Fig. 9E). Both the resting membrane potential (Fig. 9F) and rheobase (Fig. 9G) remained unchanged across the different conditions.

Collectively, our findings show that decreasing *S*-palmitoylation in human DRGs diminishes the firing of action potentials in sensory neurons. This occurs through a mechanism that entails a loss-of-function by right-shifting the voltage-dependence of activation. Interestingly, this effect contrasts with the impact of 2-BP on sodium currents observed in heterologous expression systems and rodents, suggesting a distinct mechanism of action for 2-BP in sodium channels expressed in human sensory neurons.

## Discussion

In this study, using biochemical and electrophysiological approaches, we showed for the first time that native Na_V_1.7 channels are regulated by *S*-palmitoylation. We identified two cysteines located in the intracellular loop connecting domains II and III (in loop 2)—Cys1126 and Cys1152—as Na_V_1.7 *S*-palmitoylation sites. Our findings additionally show that the *S*-palmitoylation of these cysteine residues has differential effects on Na_V_1.7 functions. Specifically, *S*-palmitoylation of Cys1126 increases Na_V_1.7 current density but does not affect its steady-state inactivation. On the other hand, *S*-palmitoylation of Cys1152 affects the channel's steady-state inactivation but does not influence Na_V_1.7 current density. Finally, pharmacological manipulation of *S*-palmitoylation reduces excitability of rat and human DRGs. Together, these findings unveil a post-translational regulatory mechanism by which *S*-palmitoylation modulates specific aspects of Na_V_1.7 function and cellular activity.

It is well established that *S*-palmitoylation regulates the trafficking of soluble proteins between the cytosol and the membrane (16). However, regulation of transmembrane proteins by this post-translational modification is more complex. Consequences of membrane protein *S*-palmitoylation include proper assembly, maturation, trafficking, association and conformation of transmembrane domains, protein–protein interactions, crosstalk with other post-translational modifications and signaling pathways, internalization, recycling, and degradation (17). Inhibiting palmitoylation by 2-BP and antagonizing Na_V_1.7 by ProTx-II caused a similar level of reduction of DRG sodium currents, which strongly indicates that *S*-palmitoylation plays a pivotal role in regulating the function of Na_V_1.7 channels. Typically, a reduction in current density suggests an influence on the membrane localization of these channels. However, it is worth noting that voltage-gated sodium channel β2 subunits, responsible for controlling the localization of Na_V_s at the plasma membrane (18, 19), are also susceptible to *S*-palmitoylation (10). *S*-palmitoylation of these auxiliary subunits enhances their association with detergent-resistant membranes (10), and the addition of 2-BP to DRG cultures may potentially affect *S*-palmitoylation of these subunits, contributing to the observed decrease in sodium current density. Importantly, the observed effects of 2-BP in HEK293 cells expressing Na_V_1.7 α1 subunit alone, suggests that *S*-palmitoylation of Na_V_1.7 α1 subunit potentially regulates Na_V_1.7 cell surface expression by directly regulating the channel's trafficking. It is important to note that the precise stage of the trafficking pathway at which *S*-palmitoylation occurs remains uncertain. For Na_V_1.2 (20) and K_V_1.5 (21, 22) channels, *S*-palmitoylation occurs at the early stages of biosynthesis, regulating the maturation of these channels. Interestingly, internalization of K_V_1.5 channels is also regulated by *S*-palmitoylation, where a higher surface expression is observed in palmitoylated-deficient channels (22).

Our data revealed no significant difference between DRGs treated with PA alone and those treated with PA and ProTx-II. This observation suggests that: (i) the effect of ProTx-II on Na_V_1.7 channels could potentially be masked, as other sodium channels might be *S*-palmitoylated when PA is present; (ii) *S*-palmitoylation affects Na_V_1.7 channel pharmacology by altering its affinity for ProTx-II at the voltage-sensor domain II (23), analogous to the observed impact on Na_V_1.2 channels (24). This hypothesis is further supported by the less pronounced effect of ProTx-II on the voltage-dependent activation in the PA-treated group. ProTx-II typically inhibits sodium currents by shifting the voltage-dependence of channel activation toward more positive potentials (25), and this effect was attenuated in the presence of both ProTx-II and PA in our experiments. Although it has been convincingly demonstrated that addition of PA facilitates membrane association, the functional consequences depend on the location of the targeted cysteines within the structure of the channel (17). Of the two cysteine residues identified in this study, only Cys1152 is highly conserved among Na_V_ isoforms. However, this was not identified as a *S*-palmitoylation site in Na_V_1.2, Na_V_1.5, or Na_V_1.6 channels (9, 15, 20, 24). This suggests that *S*-palmitoylation might not consistently occur at conserved sites and that its function varies depending on the specific Na_V_ isoform. It was found that a mutation in an intracellular loop of Na_V_1.2 (G1079C) causes an enhanced *S*-palmitoylation of these channels, resulting in a higher affinity for the tarantula toxin PaurTx3 and ProTx-II. Further investigations led to the identification of three endogenous cysteine sites in Na_V_1.2 that regulate the gating as well as the pharmacological properties of these channels (24).

Studies conducted by the Cummins laboratory showed that *S*-palmitoylation of cardiac Na_V_1.5 channels modulates the biophysical properties of the channel without significantly affecting current density.

This modification increases channel availability and, consequently, has an impact on cardiomyocyte excitability (15). For Na_V_1.6 channels, *S*-palmitoylation also regulates distinct functions by modifying different residues. Similar to what we observed in this study, both current density and voltage-dependence properties of Na_V_1.6 channels are modulated by this modification (9). Thus, the observed effects on Na_V_1.7 may be due to changes in the channel's structural conformation. However, whether this or other mechanisms are involved in the regulation of Na_V_1.7 remains to be determined.

The regulation of *S*-palmitoylation is not only dependent on the specific location of targeted cysteine residues but is influenced by a complex interplay of various factors (4). Enzyme-substrate specificity exhibited by palmitoyl acyltransferases (zDHHC-PATs) also plays an important role in this regulation (26). Furthermore, these zDHHC-PATs can be localized within specific organelles, allowing them to modify proteins at different stages of their life cycle. Notably, there are 23 identified isoforms of zDHHC-PATs in mammals (3). Previous studies have demonstrated that DRG neurons express all 23 of these mammalian zDHHC-PATs (27, 28). Moreover, protein depalmitoylation is governed by palmitoyl thioesterases to regulate the balance between palmitoylation and depalmitoylation. It has been reported that, in hippocampal neurons, ZDHHC14 controls Kv1-family potassium channel clustering at the axon initial segment, and loss of ZDHHC14 decreases outward currents and increases AP firing in hippocampal neurons (29). Additionally, zDHHCs have been shown to selectively interact with various ion channels, including GABA_A_ (30) and large conductance voltage- and calcium-activated potassium channels (31). These observations suggest the existence of multimolecular signaling complexes involving channels and palmitoylating enzymes. However, their specific role in DRG neurons has not yet been studied in detail.

*S*-palmitoylation of a number of proteins has been associated with pain in several human disorders, including cancer, diabetes, Alzheimer's disease, and cystic fibrosis (4). For instance, *S*-palmitoylation of peroxiredoxin-6, an antioxidant enzyme, enhances its interaction with anion exchanger 3 and activates the Cl^−^/HCO3^−^ flux inducing pain in diabetic neuropathy (32). Palmitoylation of the A-kinase anchoring protein 150 (33), which organizes kinases and phosphatases to regulate AMPA receptors (34), contributes to pain hypersensitivity by facilitating synaptic incorporation of GluA1-containing AMPA receptor in the spinal dorsal horn (35). *S*-palmitoylation of GluA1 regulates its membrane expression (36) and may impact synaptic plasticity (37) likely contributing to pain. In line with this, palmitoylation has shown to be involved in the internalization of the NMDA receptor 2B subunit (NR2B) (38). Chronic compression of DRGs induces upregulation of NMDA palmitoylation in the spinal cord, while intrathecal administration of 2-BP reversed pain-like behaviors and reduced palmitoylation of NR2B (38). *S*-palmitoylation also seems to contribute to depression-like behaviors induced by chronic pain. For example, spared nerve injury activated astrocytic release of interleukin 6 in the basolateral amygdala (39), which promoted palmitoylation of PSD-95 (40, 41), a protein required for proper localization of AMPAR and NMDAR in the postsynaptic density (40). *S*-palmitoylation of PSD-95 enhances the synaptic trafficking of GluA1 and NR2B, and subsequently mediates the depression-like behaviors induced by nerve injury (39). Importantly, these effects are attenuated by injection of 2-BP in bilateral basolateral amygdala area (39). This collective evidence suggests that targeting *S*-palmitoylation may be a broadly applicable strategy for pain treatment.

Na_V_1.7 is a major contributor to pain signaling (42, 43). While direct inhibition of Na_V_1.7 has not yielded effective pain treatments (44), emerging alternative approaches hold promise for a breakthrough (45–48). In DRG neurons, Na_V_1.7 channels set the threshold for action potentials by conducting subthreshold sodium currents (7). Our data show that blocking *S*-palmitoylation with 2-BP leads to a decrease of neuronal excitability in rodents and humans. Even though constitutive hDRG neuron excitability was low (control group) in our experiments, we show for the first time, that 2-BP treatment caused a reduction in the number of action potentials when compared to cells treated with PA. Basal hDRG excitability can vary among individuals, and low excitability can be influenced by several factors, including both physiological and pathological conditions of the donor. However, the decreased number of action potentials induced by 2-BP may partially stem from the observed ∼9 mV rightward shift in the voltage-dependence of activation of sodium currents when 2-BP is present. Furthermore, the voltage-dependence activation and inactivation curves cross at ∼−31.2 mV in the control condition, ∼−28 mV in the presence of PA, and ∼−23.3 mV with 2-BP, suggesting that in the absence of palmitoylation, stronger depolarization is required to activate sodium channels.

One limitation of our work is that when recording from DRG neurons which express varying amounts of Na_V_1.7, Na_V_1.8, and Na_V_1.9 channels, disrupting the activity of a specific channel by targeting its post-translational modifications could change the proportion of the remaining active channels leading to a flattening of the slope of fast inactivation in those sensory neurons. It is important to note from our recordings in Na_V_1.7 channels expressed in the heterologous system that the slope factor remains unaffected among DMSO, PA, and 2-BP groups suggesting that changes in the proportion of Na_V_ channels in DRG neurons along with changes in post-translational modifications confer alterations in their *V*_1/2_ and *k* parameters. In addition, the voltage-clamp recordings of human DRGs neurons revealed differences between the heterologous expression and hDRG data. However, this could potentially be attributed to the presence of additional ion channels, auxiliary subunits, or associated proteins in the DRGs. These components might undergo post-translational modifications at cysteine residues, which could influence the functional impact of palmitoylation and depalmitoylation events. Such modifications could control conformational changes or physical interactions within the nano environment, thereby leading to alternative regulatory mechanisms.

Another limitation of our findings is that the pharmacological treatments used (PA and 2-BP) not only affect the *S*-palmitoylation of Na_V_1.7 but also impact a wide range of proteins within sensory neurons. In this regard, there are cases in which *S*-palmitoylation seems to have a protective role. For instance, *S*-palmitoylation prevents sustained inflammation by limiting nucleotide-binding oligomerization domain, leucine-rich repeat and pyrin-domain-containing 3 (NLRP3) inflammasome activation (49). Another limitation is that, *S*-palmitoylation of other Na_V_ isoforms (4) and other ion channels, including calcium (50) and potassium channels (29), could potentially contribute to the observed effects on AP activity in DRG neurons. For instance, *S*-palmitoylation of Na_V_1.6 channels also affects current density and kinetics of these channels modulating DRG neuronal excitability (9). Furthermore, it was reported that *S*-palmitoylation of δ-catenin promotes Na_V_1.6 trafficking to the plasma membrane in DRG neurons contributing to increase transmission of nociceptive signals (11).

Collectively, our results show, for the first time, that *S*-palmitoylation has a direct impact on Na_V_1.7 channels, effectively regulating their function and thereby influencing the excitability of rodent sensory neurons. Importantly, we observed similar findings in humans, underscoring the translational relevance of our results. This work sets the stage for further studies, offering opportunities to develop precise drugs targeting *S*-palmitoylation in Na_V_1.7 channels for potential therapeutic applications, including chronic pain.

## Materials and Methods

Detailed descriptions of experiments and associated references are available in Supplementary Material, Materials and Methods. This study aimed to assess if Na_V_1.7 channels are subject to post-translational modification by *S*-palmitoylation. *S*-palmitoylation sites were computationally predicted for Na_V_1.7 and site-directed mutagenesis studies were used to pinpoint the sites targeted by this modification. Biochemical and electrophysiology experiments were performed in CAD cells and HEK293 cell lines, deidentified human spinal cord samples as well as in DRG neurons from rats and de-identified human donors. All electrophysiology and biochemistry experiments were performed according to established protocols (46, 51, 52). Animal protocols received approval from the College of Medicine's Institutional Animal Care and Use Committees of the involved Universities, aligning with the NIH's Guide for Care and Use of Laboratory Animals. We determined sample sizes from our past experiments. Researchers were blind to treatments, and groups were allocated at random.

## Supplementary Material

pgae222_Supplementary_Data

## Data Availability

All data are available in the main text, figures, Supplementary Materials, and dataset.
