# Supplementary material for: Regulating neuronal excitability: The role of S-palmitoylation in NaV1.7 activity and voltage sensitivity
Source: PNAS Nexus. 2024 Jun 4;3(6):pgae222. doi: 10.1093/pnasnexus/pgae222 (PMC11184981; doi:10.1093/pnasnexus/pgae222)
Supplement: pgae222_Supplementary_Data [file pgae222_supplementary_data.zip › PNASNEXUS-PNASNEXUS-2024-00225R-s01.docx]

­**
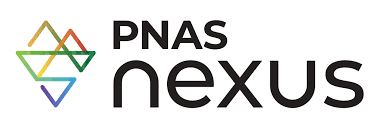
**

**Supporting Information for**

Regulating Neuronal Excitability: The Role of *S*-Palmitoylation in Na_V_1.7 Activity and Voltage Sensitivity

Cheng Tang^1-3^, Paz Duran^1,†^, Aida Calderon-Rivera^1,4,†^, Santiago Loya-Lopez^1,4,†^, Kimberly Gomez^1,4^, Samantha Perez-Miller^1,4^, and Rajesh Khanna^4^*

Affiliations:

^1^Department of Molecular Pathobiology, College of Dentistry, New York University, New York, NY, 10010, USA.

^2^The National and Local Joint Engineering Laboratory of Animal Peptide Drug Development, College of Life Sciences, Hunan Normal University, Changsha, 410081, China.

^3^Peptide and small molecule drug R&D platform, Furong Laboratory, Hunan Normal University, Changsha, 410081, China.

^4^Department of Pharmacology and Therapeutics, College of Medicine, University of Florida, Gainesville, FL 32610

^†^Contributed equally to this work

*Correspondence to:

Dr. Rajesh Khanna, Department of Pharmacology and Therapeutics, University of Gainesville College of Medicine. Office phone: (520) 271-0433; Email: [r.khanna@ufl.edu](mailto:rk4272@nyu.edu)

**This PDF file includes:**

SI Materials and Methods

Figure S1-2

Tables S1-5

SI References

**Supporting Information**

**SI Materials and Methods**

**Chemicals**

All chemicals, including 2-Bromopalmitate (2-BP) and palmitic acid (PA), were obtained from Sigma (St. Louis, MO) unless stated otherwise.

**Palmitoylation Site Prediction**

Palmitoylation sites were predicted for mouse Na_V_1.7 (UniProt Accession Q62205) using CSS-Palm-4.0 (1) using the default medium threshold. The following sites were predicted: C134, C894, C896, C934, C1163, C1164, C1257, C1728, C1836. Sites located on extracellular loop II (C894, C896), the extracellular vestibule (C934), or extracellular loop IV (C1728) were not tested as these would not be accessible to the palmitoylation machinery. Although GPS-Palm (2) is an update to CSS-Palm, CSS-Palm-4.0 was used in order to directly compare to predictions made for Na_V_1.6 by (3).

**Plasmids, site-directed mutagenesis**

The complete open reading frame of mouse NaV1.7 was cloned into the pcDNA3.1(+)-C-DYK vector. Mutations to NaV1.7 plasmid used in this study were designed in-house and purchased from GenScript. All constructs were sequenced to confirm mutation sites.

**Sequence Alignment**

The sequence alignment for Na_V_ isoforms was generated using the MAFFT-L-INS-i algorithm (4) as implemented in JalView (5, 6) using the full-length mouse sequences (Na_V_1.1, UniProt Accession A2APX8; Na_V_1.2, B1AWN6; Na_V_1.3, A2ASI5; Na_V_1.4, Q9ER60; Na_V_1.5, Q9JJV9; Na_V_1.6, Q9WTU3; Na_V_1.7, Q62205; Na_V_1.8, Q6QIY3; Na_V_1.9, Q9R053). The ~50 amino acid region at the C-terminal end of intracellular Loop 2 was extracted and is displayed using the Zappo coloring schema.

**Cell culture of mouse catecholamine A differentiated (CAD) cells and HEK293 cell lines**

Mouse neuron derived mouse catecholamine A differentiated (CAD) (ECACC cat. no. 08100805, RRID: CVCL_0199) and human-derived HEK293 cells were grown in standard cell culture conditions, 37 °C in 5% (vol/vol) CO2, as previously described (7). All media were supplemented with 10% (vol/vol) FBS (HyClone) and 1% penicillin/streptomycin sulfate from 10,000 μg/mL stock. CAD cells were maintained in DMEM/F12 media, and HEK293 cells were maintained in DMEM media. CAD cells were chosen as a model neuron cell line because of ∼80% expression of NaV1.7 channels.

HEK293 cells were transfected with 3 µg of the mouse NaV1.7 plasmids without the β subunit. For the electrophysiological recordings 0.5 µg of GFP plasmid was added. Transfection was performed using Lipofectamine 2000 (cat. no. 11668019, Thermo Fisher Scientific, Waltham, MA) according to manufacturer’s instructions. Cells were plated on glass 12 mm coverslips or cell culture dishes. All experiments were performed between 48 h and 48 h after transfection.

**Animals**

Pathogen-free, adult female Sprague-Dawley rats (75–100 g; Charles River, Wilmington, MA) were housed in the University of Arizona Laboratory Animal Research Center or New York University Kriser Dental Center Animal Facility in light-controlled (12-h light: 12-h dark cycle; lights on at 07:00 h) and temperature-controlled (23 ± 3°C) rooms, with standard rodent chow and water *ad libitum*. The Institutional Animal Care and Use Committees of the College of Medicine at the University of Arizona and the College of Dentistry at the New York University approved all experiments. All experiments and procedures were performed in accordance with the *Guide for Care and Use of Laboratory Animals* recommended by the National Institutes of Health.

**Isolation and culture of rat dorsal root ganglion neurons**

Dorsal root ganglia from all levels were dissociated as described previously (8, 9). Female Sprague-Dawley rats (100-150 g) were euthanized according to institutionally approved procedures. Briefly, lumbar DRGs were collected, trimmed at their roots, and enzymatically digested in DMEM (cat. no. 11965, Thermo Fisher Scientific, Waltham, MA) media with neutral protease (3.125 mg/mL, cat. no. LS02104, Worthington, Lakewood, NJ) and collagenase type I (5 mg/mL, cat. no. LS004194, Worthington, Lakewood, NJ) for 50 min at 37 ⁰C under gentle agitation. The dissociated DRG neurons were gently centrifuged to collect cells and resuspended in complete DRG media (DMEM containing 1% penicillin/streptomycin sulfate from 10,000 μg/mL stock, and 10% fetal bovine serum (Hyclone)). Cells were seeded on poly-D-lysine-coated 12 mm coverslips.

**Acyl biotin exchange**

Palmitoylation of NaV1.7 was detected using the previously described acyl biotin exchange (ABE) assay with minor modifications (10). Briefly, cells or spinal cord samples were lysed with 20 mM Tris·HCl, pH 7.4, 50 mM NaCl, 2 mM MgCl2, 1% (vol/vol) Nonidet P-40, 0.5% (mass/vol) sodium deoxycholate, 0.1% (mass/vol) SDS with Protease inhibitors (cat. no. B14002; Biotool), phosphatase inhibitors (cat. no. B15002, Biotool), and BitNuclease (cat. no. B16002; Biotool). Then, cell lysates or 1 mg of total protein from human spinal cord lysates were treated overnight with N-ethylmaleimide at 4°C to ensure complete cysteine alkylation. The next day, lysates were centrifuged at 13,500 g for 5 min. The soluble component was then washed by chloroform-methanol precipitation to remove excess N-ethylmaleimide. The protein interface was washed twice using methanol, and then protein was dissolved in 1% SDS in PBS. Protein sample was divided into two equal groups and treated with either Tris buffer (200 mM Tris, 1 mM of HPDP-Biotin, 0.2% Triton x-100 and protease inhibitors; pH 7.4) as a negative control, or NH_2_OH buffer (0.7 M hydroxylamine, 1 mM of HPDP-Biotin, 0.2% Triton x-100 and protease inhibitors; pH 7.4). The reaction was carried out protected from light for 1 h at room temperature. The excess chemicals were washed by chloroform-methanol precipitation and then dissolved in 1% Triton X-100, 0.2% SDS in PBS. Soluble proteins were incubated overnight at 4°C with Dynabeads M-280 Streptavidin (cat. no. 11205D, Invitrogen) to capture biotinylated proteins. Next day, beads were washed four times with 1% Triton X-100, 0.2% SDS in PBS. Biotinylated proteins were eluted by adding loading buffer containing dithiothreitol (DTT) and heated for 5 min at 95°C.

**Immunoblot preparation and analysis**

Indicated samples were loaded on 4–20% Novex gels (cat. no. XP04205BOX; Thermo Fisher Scientific, Waltham, MA). Proteins were transferred for 1 h at 100 V using TGS (25 mM Tris, pH 8.5, 192 mM glycine, 0.1% (mass/vol) SDS), 20% (vol/vol) methanol as transfer buffer to polyvinylidene difluoride (PVDF) membranes (0.45 μm; cat. no. IPFL00010; Millipore Sigma, St. Louis, MO), preactivated in pure methanol. After transfer, the membranes were blocked at room temperature for 1 h with TBST (50 mM Tris·HCl, pH 7.4, 150 mM NaCl, 0.1% Tween 20) with 5% (mass/vol) nonfat dry milk, and then incubated separately in indicated primary antibodies NaV1.7 (cat. no. ab85015; RRID:AB_2184346; Abcam, Cambridge, United Kingdom), GAPDH (cat. no. TA802519; RRID:AB_2626378; Origene, Rockville, MD), in TBST, 5% (mass/vol) BSA, overnight at 4°C. Following incubation in HRP-conjugated secondary antibodies from Jackson Immuno Research (West Grove, PA) (1/10,000 dilution), Mouse Anti-Rabbit (cat. no. 211-032-171, RRID:AB_2339149) and Goat Anti-Mouse (cat. no. 115-035-174, RRID:AB_2338512), blots were revealed by enhanced luminescence (cat. no. WBKLS0500; Millipore Sigma, St. Louis, MO) before exposure to photographic film as described (11).

**Patch-clamp electrophysiology**

All recordings were done using procedures adapted from our prior work (7, 9, 12, 13).

For DRG recordings, acutely dissociated DRG neurons from Sprague Dawley rats with a capacitance value below 30 pF, which has been historically associated with the population of small diameter DRG neurons, were used (14).

Voltage-clamp recordings of rat DRG neurons

For sodium current recordings, the internal pipette solution consisted of (in mM): 140 CsF, 10 NaCl, 1.1 Cs-EGTA, and 15 HEPES (pH 7.3, mOsm/L = 290-310) and external solution contained (in mM): 50 NaCl, 100 tetraethylammonium chloride, 10 D-glucose, 1.8 CaCl_2_, 0.1 CdCl_2_, 1 MgCl2, and 10 HEPES (pH 7.3, mOsm/L = 310-315). DRG neurons were interrogated with current-voltage (I-V) and activation/inactivation voltage protocols. The voltage protocols were as follows: (i) I-V protocol: from a holding potential of −60 mV, cells were depolarized with 150-millisecond voltage steps over a range of −70 to +60 mV in +5 - mV increments; (ii) inactivation protocol: from a holding potential of −60 mV, cells were subjected to hyperpolarizing/repolarizing pulses for 1 second over a range of −120 to 0 mV in +10 mV steps, followed by a 200-millisecond test pulse to +10 mV.

Inactivation curves were obtained by dividing the current amplitude recorded at the test pulse by the maximum current (Imax). Activation and SSI curves were fitted with the Boltzmann equation.

In experiments utilizing Br-palmitate (2BP) and palmitic acid (PA), the compounds were incubated for 24 h at a final concentration of 25 μM and 10 μM respectively. When Na_V_1.7-blocker was employed, ProTx-II was added into the external recording solution at a final concentration of 5 nM.

Normalization of currents to each cell’s capacitance (pF) was performed to collect the current density data. For I-V curves, functions were fitted to data using a non-linear least squares analysis. I-V curves were fitted using double Boltzmann functions:

*f = a+ g1/(1+exp((x-V_1/2_1)/k1)) + g2/(1+exp(-(x-V_1/2_2)/k2))*

where *x* is the pre-pulse potential, *V_1/2_* is the mid-point potential and *k* is the corresponding slope factor for single Boltzmann functions. Double Boltzmann fits were used to describe the shape of the curve, not to imply the existence of separate channel populations. Numbers *1* and *2* simply indicate first and second mid-points; *a* along with *g* are fitting parameters.

Activation curves were obtained from the I-V curves by dividing the peak current at each depolarizing step by the driving force according to the equation: *G = I/(V_mem_-E_rev_)*, where *I* is the peak current, *V_mem_* is the membrane potential and *E_rev_* is the reversal potential. The conductance (*G*) was normalized against the maximum conductance (*G_max_*). Activation curves were fitted with the Boltzmann equation:

G/Gmax=1/[1+*exp*(V_0.5_−Vm)/*k*],

where *G* is the conductance in *G=I*/(*V*_m_ – *E*_rev_), *G*_max_ the maximal conductance obtained from the Boltzmann fit under control conditions, *V*_0.5_ the voltage for half-maximal activation or inactivation, *V*_m_ the membrane potential, and *k* a slope factor.

Inactivation curves were obtained by dividing the peak current recorded at the test pulse by the maximum current (*I_max_*). Steady state inactivation (SSI) was fitted with the equation:

*I/I*_max_=1/[1+exp(V_m_−V_0.5_)/*k*]

where *I* is the current, *I_max_* the maximal current obtained from the Boltzmann fit under control conditions, *V*_0.5_ the voltage for half-maximal activation or inactivation, *V_m_* the membrane potential, and *k* a slope factor.

The reversal potential for *I*_Na_^+^ (*E*_rev_) was determined for each individual neuron. Capacitive artifacts were fully compensated, and series resistance was compensated by ∼70 %. Recordings made from cells with greater than a 20% shift in series resistance compensation error were excluded from the analysis. All experiments were performed at room temperature (∼23 °C).

Current-clamp recordings of rat DRG neurons

Whole-cell current-clamp experiments were carried out on rat DRG sensory neurons using a HEKA EPC-10 patch-clamp amplifier (HEKA Elektronik, Lambrecht, Germany). Borosilicate glass capillaries with resistances between 2.5 and 3.5 MΩ were filled with internal solution containing 120 mM K-Gluconate, 10 mM NaCl, 4 mM Mg-ATP, 5 mM EGTA and 10 mM HEPES, 2 mM MgCl_2_ (Osmolarity= 290 mOsm, pH= 7.3 adjusted with KOH). Action potentials (AP) were evoked in response to depolarizing current injections of 0-120 pA with an increment of 10 pA in 300 ms in the presence of 0.1 % DMSO, PA or 2BP. The bath recording solution contained 140 mM NaCl, 4 mM KCl, 2 mM CaCl_2_, 2 mM MgCl_2_, 10 mM glucose and 10 mM HEPES (Osmolarity= 310 mOsm, pH= 7.4).

Rheobase was measured by injecting currents from 0 pA with an increment of 10 pA in 100 ms. DRG neurons with a resting membrane potential (RMP) more hyperpolarized than −40 mV, stable baseline recordings, and evoked spikes that overshot 0 mV were used for experiments and analysis.

Voltage-clamp recordings of HEK293 cells

Transiently-transfected HEK 293 cells were subjected to current-voltage (I-V) and activation/inactivation voltage protocols as follows: a) for the I-V protocol, cells were held at a potential of −110 mV and depolarized by 150-ms voltage steps from −70 mV to +60 mV in 5-mV increments, the resulting currents were normalized to the cell size, expressed as cell capacitance in pF, to get the corresponding current density and also infer the peak current density. The activation voltage dependence of sodium channels was analyzed as a function of current versus voltage; b) inactivation protocol: from a holding potential of −110 mV, cells were subjected to 1-s hyperpolarizing/repolarizing pulses between−120 and 10 mV (+10 mV steps) followed by a 200-ms test pulse to +20 mV. This increase in membrane potential conditioned various proportions of sodium channels into a state of fast inactivation. The external solution for voltage-clamp sodium recordings contained 140 mM NaCl, 30 mM tetraethylammonium chloride, 10 mM D-glucose, 3 mM KCl, 1 mM CaCl2, 0.5 mM CdCl2, 1 mM MgCl2, and 10 mM HEPES (pH 7.3 and 310-315 mOsm); and the internal solution consisted of: 140 mM CsF, 10 mM NaCl, 1.1 mM Cs-EGTA, and 15 mM HEPES (pH 7.3 and 290-310 mOsm). Analysis was performed by using Fitmaster software.

To determine the recovery from fast inactivation, from a holding potential of −90 mV, cells were subjected to two 50-ms hyperpolarizing test pulses (t1 and t2) to 0 mV separated by a repolarizing pulse to -90 mV for increasing recovery durations (Δt; 0 ms, 1 ms, 2 ms, 4 ms, 8 ms, 16 ms, 32 ms, 64 ms, 128 ms, 256 ms, 512 ms, or 1024 ms). Fraction recovered is calculated as I_t2_/I_t1_.

To determine the voltage-dependent development of slow inactivation, from a holding potential of −90 mV, cells were subjected to two 50-ms hyperpolarizing test pulses (t1 and t2) to 0 mV separated by conditional pulses from -120 mV to +40 mV for 8 s (in +10 mV increments) to develop slow inactivation. Fast inactivation was almost fully recovered by clamping the channels at -90 mV for 100 ms after t1 and before t2; I_t2_/I_t1_ represents the proportion of channels remaining not inactivated after the conditional pulse clamping.

**Human dorsal root ganglion neuron cultures**

All human tissue procurement procedures were approved by the Institutional Review Boards at New York University. hDRG suspension cells were obtained from AnaBios Corporation. Donor information is provided in **supplementary table 3**. Cells were recovered with a gentle centrifugation (~350 xG ) at room temperature for 3 min and the pellet was gently resuspend with 2 mL of complete DMEM/F12 media containing 1% penicillin/streptomycin sulfate (Cat# 15140, Life Technologies, Carlsbad, CA), 10% horse serum (Cat#SH3007403, Cytiva HyClone, Logan, UT) 25 ng/mL hNGF (Cat#256GF100CF, Fisher scientific, Pittsburgh, PA), and 25 ng/mL GDNF (Cat#RP-8602, Thermo fisher, Waltham, MA). The cells were seeded on poly-D-lysine (0.1 mg/ml; Cat# P6407, Millipore Sigma, St. Louis, MO) and laminin (1 mg/ml; Cat#sc-29012, Santa Cruz Biotechnology, Dallas, TX)-coated 12-mm glass coverslips and incubated at 37°C. Half of the culture media is replaced with fresh media every 3 days. All cultures were used within 96 hours.

**Whole-cell patch-clamp recordings of sodium currents and evoked action potentials in acutely dissociated hDRG neurons**

For voltage-clamp recordings, we utilized identical solutions and protocols to those employed for rat DRG neurons.

For current-clamp recordings the external solution contained (in millimolar): 130 NaCl, 3 KCl, 2.5 CaCl_2_, 0.6 MgCl_2_, 10 D-Glucose, and 10 HEPES (pH 7.4 adjusted with NaOH, and mOsm/L= 325). The internal solution was composed of (in millimolar): 110 K-methanesulfonate, 30 KCl, 5 NaCl, 1 CaCl_2_, 2 MgCl_2_, 2 Mg-ATP, 1 Li-ATP, 11 EGTA, and 10 HEPES (pH 7.4 adjusted with KOH, and mOsm/L= 310) (15). At room temperature (22–24°C), whole-cell patch clamp configuration was made, and current-clamp mode was performed to record action potentials. DRG neurons with a resting membrane potential (RMP) more hyperpolarized than −40 mV, stable baseline recordings, and evoked spikes that overshot 0 mV were used for experiments and analysis. The action potentials were evoked by a ramp pulse from 0–1000 pA in 1 sec. Rheobase was measured by injecting currents from 0 pA with an increment of 50 pA in 100 ms. Analyses were performed by using Fitmaster software (HEKA) and Origin 9.0 software (OriginLab).

**Data Analysis**

All data plotted represent mean ± SEM. Statistical analysis and graphs were performed with GraphPad Prism version 9.5. All data sets were checked for normality using D’Agostino & Pearson test. Details of statistical tests, significance and sample sizes are reported in the appropriate figure legends and in **Dataset 1**. In western blots, *n* is presented as the number of separate experiments, statistical differences were determined by one-way ANOVA with Tukey’s post hoc test. For electrophysiological recordings, when more than two groups were compared, peak current densities were analyzed using one-way ANOVA with Tukey’s post hoc test. When two groups were compared, peak current densities were analyzed by unpaired t-test. *V_1/2_* midpoint potentials and *k* slope factors were compared using one-way ANOVA with Tukey’s post hoc test and unpaired t-test.

**SI Figures and Legends.**

**
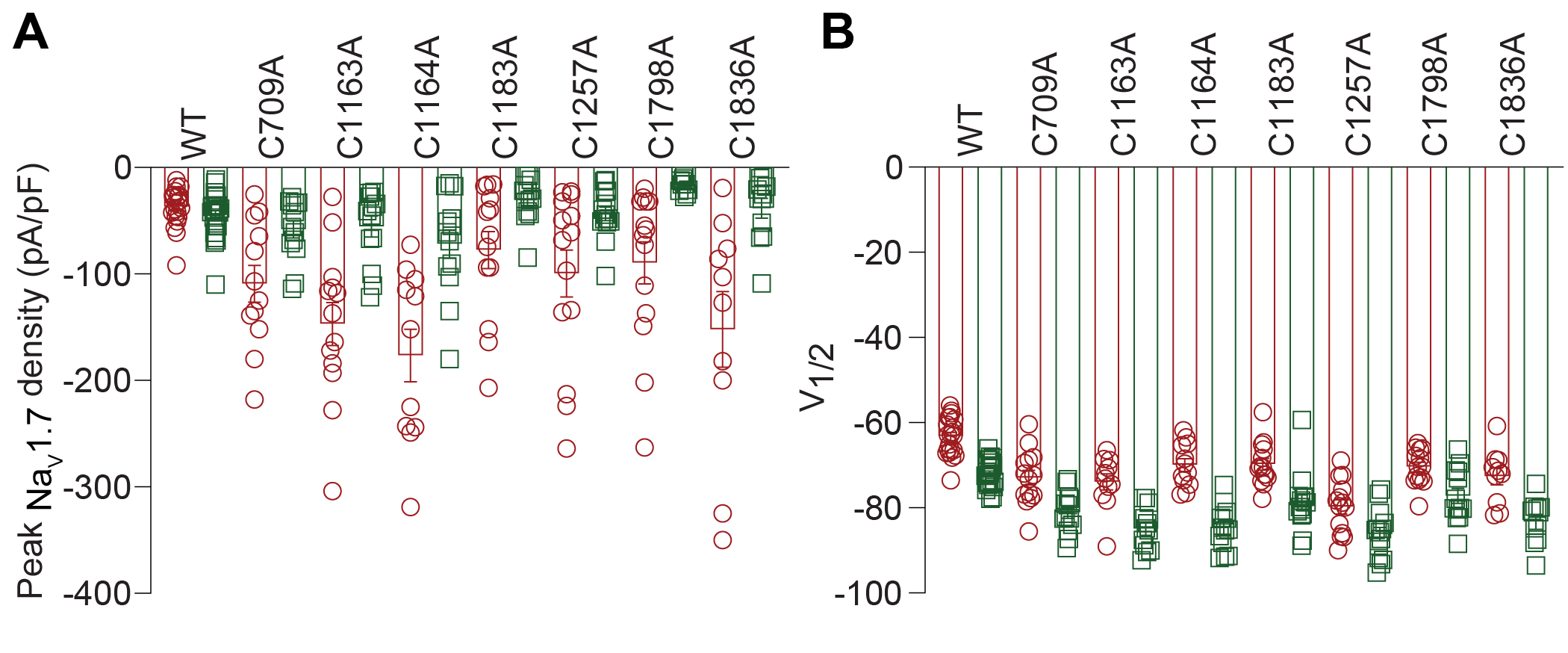
**

**Figure S1**. Summary of peak Na_V_1.7 current density (A) and half-maximal inactivation potential (V_1/2_) (B) for Na_V_1.7-WT and those Na_V_1.7 mutant channels carrying the non-critical cysteine mutations, channels were expressed in HEK293 cells and treated overnight with 10 μM PA (red circles) to enhance *S*-palmitoylation, or 25 μM 2-BP (green squares) to block *S*-palmitoylation. Data are presented as mean ± SEM. For full statistical analyses, see **Dataset 1.**


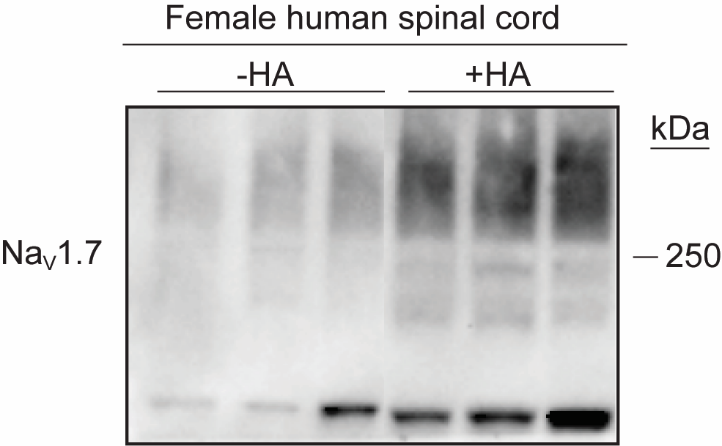


**Figure S2****. *S*-palmitoylation is present in female human spinal cord.** Representative immunoblots of ABE assay of spinal cord samples from female human donors (n=3; **Table S3**).

**SI Tables.**

**Table S1.** Gating properties of voltage-gated sodium channels in rat dorsal root ganglion neurons.

|  |  |  | Activation | | | Inactivation | | |
| --- | --- | --- | --- | --- | --- | --- | --- | --- |
|  | **Peak *I* density**  **(pA/pF)** | **n** | ***V_1/2_***  **(mV)** | ***k*** | **n** | ***V_1/2_***  **(mV)** | ***k*** | **n** |
| DMSO | -308.29 ± 29.06 | 25 | -17.73 ± 0.42 | 4.93 ± 0.36 | 25 | -48.48 ± 0.95 | -11.28 ± 0.89 | 24 |
| DMSO + ProTx-II | -195.82 ± 24.05 | 26 | -11.58 ± 0.68 | 6.78 ± 0.60 | 26 | -40.46 ± 1.33 | -14.26 ± 1.29 | 25 |
| PA | -304.00 ± 30.44 | 17 | -16.78 ± 0.65 | 5.68 ± 0.58 | 18 | -46.95 ± 1.04 | -12.06 ± 0.99 | 18 |
| PA + ProTx-II | -203.35 ± 27.92 | 23 | -13.12 ± 0.54 | 6.18 ± 0.47 | 23 | -47.36 ± 1.58 | -14.33 ± 1.58 | 22 |
| 2-BP | -167.83 ± 29.12 | 23 | -15.01 ± 0.72 | 6.51 ± 0.64 | 23 | -55.41 ± 2.04 | -18.86 ± 2.28 | 22 |
| 2-BP + ProTx-II | -103.01 ± 18.81 | 14 | -12.93 ± 1.59 | 10.13 ± 1.44 | 15 | -48.32 ± 3.04 | -18.99 ± 3.48 | 14 |

Values are means ± SEM. Gating properties were calculated from fits of the data from the indicated number of individual cells (in parentheses) to the Boltzmann equation; *V_1/2_* midpoint potential (mV) for voltage-dependent of activation or inactivation; *k*, slope factor. These values pertain to Figure 1. DRG, dorsal root ganglia; DMSO, dimethyl sulfoxide; ProTx-II, Protoxin-II; PA, palmitic acid; 2-BP, 2-Bromopalmitate. The statistical analyses are shown in Dataset 1.

**Table S2.** Biophysical properties of Na_V_1.7 channels in HEK293 cells

|  |  |  | Activation | | | Inactivation | | |
| --- | --- | --- | --- | --- | --- | --- | --- | --- |
|  | **Peak *I* density**  **(pA/pF)** | **n** | ***V_1/2_***  **(mV)** | ***k*** | **n** | ***V_1/2_***  **(mV)** | ***k*** | **n** |
| Na_V_1.7-WT | |  |  |  |  |  |  |  |
| DMSO | -44.63 ± 4.29 | 24 | -20.58 ± 0.43 | 4.95 ± 0.36 | 24 | -62.49 ± 0.28 | -6.02 ± 0.25 | 24 |
| PA | -36.95 ± 3.77 | 22 | -20.90 ± 0.36 | 4.34 ± 0.31 | 22 | -63.23 ± 0.38 | -6.38 ± 0.33 | 21 |
| 2-BP | -23.72 ± 2.19 | 24 | -21.51 ± 0.32 | 5.23 ± 0.27 | 24 | -72.03 ± 0.30 | -6.21 ± 0.26 | 23 |
| Na_V_1.7-C1126A/C1152A | |  |  |  |  |  |  |  |
| DMSO | -65.40 ± 9.79 | 21 | -20.19 ± 0.79 | 7.41 ± 0.70 | 19 | -85.37 ± 0.98 | -9.30 ± 0.85 | 19 |
| PA | -68.50 ± 7.14 | 31 | -20.28 ± 0.43 | 7.25 ± 0.38 | 29 | -87.92 ± 0.95 | -10.07 ± 0.80 | 28 |
| 2-BP | -48.74 ± 5.77 | 27 | -20.68 ± 0.66 | 7.80 ± 0.59 | 27 | -89.45 ± 0.99 | -9.88 ± 0.82 | 26 |
| Na_V_1.7-C709A | |  |  |  |  |  |  |  |
| PA | -109.20 ± 17.23 | 12 | -20.17 ± 0.57 | 6.05 ± 0.50 | 10 | -73.45 ± 0.74 | -9.38 ± 0.66 | 14 |
| 2-BP | -58.25 ± 7.99 | 13 | -19.02 ± 0.35 | 6.91 ± 0.31 | 12 | -81.30 ± 0.57 | -9.05 ± 0.49 | 13 |
| Na_V_1.7-C1126A | |  |  |  |  |  |  |  |
| PA | -35.74 ± 5.12 | 9 | -21.79 ± 1.31 | 6.75 ± 1.17 | 11 | -76.30 ± 0.57 | -7.70 ± 0.49 | 12 |
| 2-BP | -30.36 ± 5.21 | 11 | -21.12 ± 0.66 | 6.55 ± 0.59 | 10 | -81.45 ± 0.94 | -10.29 ± 0.81 | 8 |
| Na_V_1.7-C1152A | |  |  |  |  |  |  |  |
| PA | -78.99 ± 20.04 | 12 | -23.71 ± 0.42 | 7.65 ± 0.39 | 12 | -87.62 ± 0.54 | -7.52 ± 0.46 | 10 |
| 2-BP | -31.67 ± 6.21 | 11 | -27.57 ± 0.36 | 6.53 ± 0.32 | 10 | -90.47 ± 0.85 | -8.00 ± 0.71 | 11 |
| Na_V_1.7-C1163A | |  |  |  |  |  |  |  |
| PA | -147.00 ± 20.26 | 13 | -21.31 ± 0.69 | 6.90 ± 0.62 | 11 | -73.31 ± 0.61 | -7.30 ± 0.53 | 11 |
| 2-BP | -55.39 ± 9.73 | 13 | -21.54 ± 0.36 | 7.22 ± 0.32 | 12 | -85.15 ± 0.58 | -8.01 ± 0.49 | 12 |
| Na_V_1.7-C1164A | |  |  |  |  |  |  |  |
| PA | -176.50 ± 24.65 | 11 | -26.40 ± 0.43 | 5.09 ± 0.37 | 10 | -70.09 ± 0.52 | -8.07 ± 0.46 | 12 |
| 2-BP | -71.84 ± 13.41 | 13 | -25.94 ± 0.42 | 6.12 ± 0.37 | 11 | -84.70 ± 0.60 | -8.30 ± 0.51 | 13 |
| Na_V_1.7-C1183A | |  |  |  |  |  |  |  |
| PA | -77.54 ± 17.32 | 13 | -16.36 ± 0.64 | 7.19 ± 0.57 | 14 | -69.73 ± 0.60 | -9.10 ± 0.53 | 14 |
| 2-BP | -30.35 ± 5.76 | 13 | -17.80 ± 0.66 | 7.90 ± 0.60 | 14 | -79.61 ± 0.69 | -8.84 ± 0.60 | 14 |
| Na_V_1.7-C1257A | |  |  |  |  |  |  |  |
| PA | -99.57 ± 21.96 | 14 | -25.13 ± 0.41 | 6.25 ± 0.36 | 14 | -80.35 ± 0.72 | -8.65 ± 0.63 | 14 |
| 2-BP | -43.39 ± 6.31 | 14 | -24.89 ± 0.49 | 6.84 ± 0.44 | 14 | -86.34 ± 0.70 | -9.54 ± 0.58 | 14 |
| Na_V_1.7-C1798A | |  |  |  |  |  |  |  |
| PA | -89.61 ± 19.71 | 14 | -13.85 ± 0.59 | 7.20 ± 0.53 | 12 | -70.21 ± 0.56 | -8.99 ± 0.49 | 14 |
| 2-BP | -17.05 ± 2.06 | 10 | -18.19 ± 0.60 | 7.76 ± 0.54 | 13 | -77.03 ± 0.70 | -8.36 ± 0.61 | 13 |
| Na_V_1.7-C1836A | |  |  |  |  |  |  |  |
| PA | -152.10 ± 35.44 | 10 | -22.65 ± 0.68 | 5.08 ± 0.60 | 8 | -72.98 ± 0.78 | -8.17 ± 0.69 | 10 |
| 2-BP | -38.17 ± 9.44 | 11 | -17.27 ± 0.71 | 7.83 ± 0.65 | 10 | -83.91 ± 0.82 | -9.11 ± 0.69 | 10 |

Values are means ± SEM. Gating properties were calculated from fits of the data from the indicated number of individual cells (in parentheses) to the Boltzmann equation; *V_1/2_* midpoint potential (mV) for voltage-dependent of activation or inactivation; *k*, slope factor. These values pertain to Figures 2, 4, 6 and S1. DMSO, dimethyl sulfoxide; PA, palmitic acid; 2-BP, 2-Bromopalmitate.

**Table S3.** Human spinal cord donor information

| AnaBios ID | Age | Sex | Ethnicity | Cause of death |
| --- | --- | --- | --- | --- |
| 211217ScHA | 57 | F | Caucasian | CVA/Stroke/ICH |
| 191204ScHA | 56 | F | Caucasian | Anoxia/Cardiovascular |
| 191206ScHA | 56 | F | Hispanic | Head Trauma/Blunt Injury |

**Table S4.** Human DRG donor information

| AnaBios ID | Age | Sex | Ethnicity | Cause of death |
| --- | --- | --- | --- | --- |
| 230927DHA | 34 | M | Filipino | Cerebral vascular accident / Intracerebral hemorrhage / Stroke |
| 231006DHA | 32 | F | African American | Anoxia / Cardiovascular |
| 231101DHA | 47 | M | Caucasian | Cerebral vascular accident / Intracerebral hemorrhage / Stroke |
| 231119DHA | 35 | F | African American | Head Trauma / Blunt Injury |

**Table S5.** Gating properties of voltage-gated sodium channels in human dorsal root ganglion neurons.

|  |  |  | Activation | | | Inactivation | | |
| --- | --- | --- | --- | --- | --- | --- | --- | --- |
|  | **Peak *I* density**  **(pA/pF)** | **n** | ***V_1/2_***  **(mV)** | ***k*** | **n** | ***V_1/2_***  **(mV)** | ***k*** | **n** |
| DMSO | -103.5 ± 21.78 | 9 | -27.52 ± 1.26 | 6.23 ± 1.14 | 9 | -38.41 ± 3.17 | -16.76 ± 2.96 | 8 |
| PA | -137.1 ± 16.55 | 7 | -24.12 ± 0.77 | 4.74 ± 0.68 | 7 | -39.76 ± 7.56 | -24.68 ± 7.89 | 7 |
| 2-BP | -123.1 ± 11.62 | 9 | -18.48 ± 0.92 | 5.2 ± 0.81 | 9 | -33.72 ± 6.45 | -23.51 ± 5.74 | 8 |

Values are means ± SEM. Gating properties were calculated from fits of the data from the indicated number of individual cells (in parentheses) to the Boltzmann equation; *V_1/2_* midpoint potential (mV) for voltage-dependent of activation or inactivation; *k*, slope factor. DMSO, dimethyl sulfoxide; PA, palmitic acid; 2-BP, 2-Bromopalmitate.

**SI References**

1. J. Ren *et al.*, CSS-Palm 2.0: an updated software for palmitoylation sites prediction. *Protein Eng Des Sel* **21**, 639-644 (2008).

2. W. Ning *et al.*, GPS-Palm: a deep learning-based graphic presentation system for the prediction of S-palmitoylation sites in proteins. *Brief Bioinform* **22**, 1836-1847 (2021).

3. Y. Pan, Y. Xiao, Z. Pei, T. R. Cummins, S-Palmitoylation of the sodium channel Nav1.6 regulates its activity and neuronal excitability. *J Biol Chem* **295**, 6151-6164 (2020).

4. K. Katoh, D. M. Standley, MAFFT multiple sequence alignment software version 7: improvements in performance and usability. *Mol Biol Evol* **30**, 772-780 (2013).

5. A. M. Waterhouse, J. B. Procter, D. M. Martin, M. Clamp, G. J. Barton, Jalview Version 2--a multiple sequence alignment editor and analysis workbench. *Bioinformatics* **25**, 1189-1191 (2009).

6. P. V. Troshin *et al.*, JABAWS 2.2 distributed web services for Bioinformatics: protein disorder, conservation and RNA secondary structure. *Bioinformatics* **34**, 1939-1940 (2018).

7. E. T. Dustrude *et al.*, Hierarchical CRMP2 posttranslational modifications control NaV1.7 function. *Proc Natl Acad Sci U S A* **113**, E8443-E8452 (2016).

8. L. Francois-Moutal *et al.*, Inhibition of the Ubc9 E2 SUMO-conjugating enzyme-CRMP2 interaction decreases NaV1.7 currents and reverses experimental neuropathic pain. *Pain* **159**, 2115-2127 (2018).

9. A. Moutal *et al.*, SARS-CoV-2 spike protein co-opts VEGF-A/neuropilin-1 receptor signaling to induce analgesia. *Pain* **162**, 243-252 (2021).

10. J. Wan, A. F. Roth, A. O. Bailey, N. G. Davis, Palmitoylated proteins: purification and identification. *Nat Protoc* **2**, 1573-1584 (2007).

11. R. Khanna *et al.*, Targeting the CaValpha-CaVbeta interaction yields an antagonist of the N-type CaV2.2 channel with broad antinociceptive efficacy. *Pain* **160**, 1644-1661 (2019).

12. A. D. Piekarz *et al.*, CRMP-2 peptide mediated decrease of high and low voltage-activated calcium channels, attenuation of nociceptor excitability, and anti-nociception in a model of AIDS therapy-induced painful peripheral neuropathy. *Mol Pain* **8**, 54 (2012).

13. J. Y. Xie *et al.*, Sustained relief of ongoing experimental neuropathic pain by a CRMP2 peptide aptamer with low abuse potential. *Pain* **157**, 2124-2140 (2016).

14. A. I. Basbaum, D. M. Bautista, G. Scherrer, D. Julius, Cellular and molecular mechanisms of pain. *Cell* **139**, 267-284 (2009).

15. J. E. Hartung *et al.*, Voltage-gated calcium currents in human dorsal root ganglion neurons. *Pain* **163**, e774-e785 (2022).
