# Supplementary material for: Regulating neuronal excitability: The role of S-palmitoylation in NaV1.7 activity and voltage sensitivity
Source: PNAS Nexus. 2024 Jun 4;3(6):pgae222. doi: 10.1093/pnasnexus/pgae222 (PMC11184981; doi:10.1093/pnasnexus/pgae222)
Supplement: pgae222_Supplementary_Data [file pgae222_supplementary_data.zip › PNASNEXUS-PNASNEXUS-2024-00225R-s02.pdf]

Dataset 1. Details of statistical analyses for all figures.

Figure panel	Assay	Statistical test; findings	Post-hoc analysis (adjusted p-value)	Number of subjects
Figure 1D	Peak total sodium current density	One-way ANOVA p<0.0001	Holm-Sidak's multiple comparisons Test<br>0.1% DMSO vs 0.1% DMSO + ProTx-II: p=0.0247<br>0.1% DMSO vs PA (10 μM): p=0.9739<br>0.1% DMSO vs 2-BP (25 μM): p=0.0032<br>0.1% DMSO vs PA (10 μM) + ProTx-II: p=0.0545<br>0.1% DMSO vs 2-BP (25 μM) + ProTx-II: p<0.0001<br>0.1% DMSO + ProTx-II vs PA (10 μM): p=0.0702<br>0.1% DMSO + ProTx-II vs PA (10 μM) + ProTx-II: p=0.9739<br>0.1% DMSO + ProTx-II vs 2-BP (25 μM): p=0.8327<br>0.1% DMSO + ProTx-II vs 2-BP (25 μM) + ProTx-II: p=0.1752<br>2-BP (25 μM) vs PA (10 μM): p=0.0148<br>2-BP (25 μM) vs 2-BP (25 μM) + ProTx-II: p=0.5297<br>2-BP (25 μM) vs PA (10 μM) + ProTx-II: p=0.8230	0.1% DMSO (n=25)<br>0.1% DMSO + ProTx-II (n=26)<br>PA (10 μM) (n=17)<br>PA (10 μM) + ProTx-II (n=23)<br>2-BP (25 μM) (n=23)<br>2-BP (25 μM) + ProTx-II (n=14)

			PA (10 µM) vs PA (10 µM) + ProTx-II:<br>p=0.1207<br>PA (10 µM) vs 2-BP (25 µM) + ProTx-II:<br>p=0.0004<br>2-BP (25 µM) + ProTx-II vs PA (10 µM) + ProTx-II:<br>p=0.1515	
Figure 1E	$V_{1/2}$ activation	One-way ANOVA<br>P<0.0001	Holm-Sidak's multiple comparisons Test<br>0.1% DMSO vs DMSO + ProTx-II:<br>p<0.0001<br>0.1% DMSO vs PA (10 µM):<br>p=0.64<br>0.1% DMSO vs PA (10 µM) + ProTx: p=0.0001<br>0.1% DMSO vs 2BP (25 µM):<br>p=0.0609<br>0.1% DMSO vs 2BP (25 µM) + ProTx: p=0.0007	0.1% DMSO (n=25)<br>0.1% DMSO + ProTx-II (n=26)<br>PA (10 µM) (n=17)<br>PA (10 µM) + ProTx-II (n=23)<br>2-BP (25 µM) (n=23)<br>2-BP (25 µM) + ProTx-II (n=14)
	k activation	One-way ANOVA<br>P<0.0001	Holm-Sidak's multiple comparisons Test<br>0.1% DMSO vs DMSO + ProTx-II:<br>p=0.287<br>0.1% DMSO vs PA (10 µM):<br>p=0.949<br>0.1% DMSO vs PA (10 µM) + ProTx: p=0.756<br>0.1% DMSO vs 2BP (25 µM):<br>p=0.511<br>0.1% DMSO vs 2BP (25 µM) + ProTx: p<0.0001	0.1% DMSO (n=25)<br>0.1% DMSO + ProTx-II (n=26)<br>PA (10 µM) (n=17)<br>PA (10 µM) + ProTx-II (n=23)<br>2-BP (25 µM) (n=23)<br>2-BP (25 µM) + ProTx-II (n=14)
	$V_{1/2}$ inactivation	One-way ANOVA<br>P<0.0001	Holm-Sidak's multiple comparisons Test	0.1% DMSO (n=25)<br>0.1% DMSO + ProTx-II (n=26)<br>PA (10 µM) (n=17)

			0.1% DMSO vs DMSO + ProTx-II: p=0.0038<br>0.1% DMSO vs PA (10 µM): p=0.986<br>0.1% DMSO vs PA (10 µM) + ProTx: p=0.9909<br>0.1% DMSO vs 2BP (25 µM): p=0.022<br>0.1% DMSO vs 2BP (25 µM) + ProTx: p=0.99<br>0.1% DMSO + ProTx vs 2BP (25 µM) + ProTx: p=0.022	PA (10 µM) + ProTx-II (n=23)<br>2-BP (25 µM) (n=23)<br>2-BP (25 µM) + ProTx-II (n=14)
	k inactivation	One-way ANOVA p=0.0074	Holm-Sidak's multiple comparisons Test<br>0.1% DMSO vs DMSO + ProTx-II: p=0.7751<br>0.1% DMSO vs PA (10 µM): p=0.9847<br>0.1% DMSO vs PA (10 µM) + ProTx: p=0.775<br>0.1% DMSO vs 2BP (25 µM): 0.0234<br>0.1% DMSO vs 2BP (25 µM) + ProTx: p=0.062	0.1% DMSO (n=25)<br>0.1% DMSO + ProTx-II (n=26)<br>PA (10 µM) (n=17)<br>PA (10 µM) + ProTx-II (n=23)<br>2-BP (25 µM) (n=23)<br>2-BP (25 µM) + ProTx-II (n=14)
Figure 2C	Peak $Na_V1.7$ current density	One-way ANOVA<br>p= 0.0003	Tukey's multiple comparisons Test<br>0.1% DMSO vs PA (10 µM): p=0.2832<br>0.1% DMSO vs 2-BP (25 µM): p= 0.0002<br>PA (10 µM) vs 2-BP (25 µM): p= 0.0277	0.1% DMSO (n=24)<br>PA (10 µM) (n=22)<br>2-BP (25 µM) (n=24)
Figure 2G	Time constant (tau) of recovery from inactivation	Unpaired t test	PA (10 µM) vs 2-BP (25 µM): p=0.0076	PA (10 µM) (n=12)<br>2-BP (25 µM) (n=14)
Figure 2I	Plateau ratio of slow inactivation	Unpaired t test	PA (10 µM) vs 2-BP (25 µM): p=0.0045	PA (10 µM) (n=11)<br>2-BP (25 µM) (n=13)

Figure 3B	Normalized *S*-palmitoylated $Na_V1.7$	One-way ANOVA p=0.0134	Holm-Sidak's multiple comparisons Test<br>0.1% DMSO vs PA (10 μM): p=0.3275<br>0.1% DMSO vs 2-BP (25 μM): p= 0.0689<br>PA (10 μM) vs 2-BP (25 μM): p= 0.0137	0.1% DMSO (n=6)<br>PA (10 μM) (n=6)<br>2-BP (25 μM) (n=6)
Figure 3D	Normalized *S*-palmitoylated $Na_V1.7$	One-way ANOVA p=0.0108	Tukey's multiple comparisons Test<br>0.1% DMSO vs PA (10 μM): p=0.2132<br>0.1% DMSO vs 2-BP (25 μM): p= 0.2176<br>PA (10 μM) vs 2-BP (25 μM): p= 0.0081	0.1% DMSO (n=6)<br>PA (10 μM) (n=6)<br>2-BP (25 μM) (n=6)
Figure 4C	Peak $Na_V1.7$ current density	Unpaired t test	PA (10 μM) vs 2-BP (25 μM)<br>C1126A: p=0.4762	C1126A: PA (10 μM) (n=9)<br>2-BP (25 μM) (n=11)
Figure 4D	$V_{1/2}$ of activation	Unpaired t test	PA (10 μM) vs 2-BP (25 μM)<br>C1126A: p= 0.6630	C1126A: PA (10 μM) (n=11)<br>2-BP (25 μM) (n=10)
Figure 4E	$V_{1/2}$ of inactivation	Unpaired t test	PA (10 μM) vs 2-BP (25 μM)<br>C1126A: p= 0.0004	C1126A: PA (10 μM) (n=12)<br>2-BP (25 μM) (n=8)
Figure 4E	*k*	Unpaired t test	PA (10 μM) vs 2-BP (25 μM)<br>C1126A: p= 0.0095	C1126A: PA (10 μM) (n=12)<br>2-BP (25 μM) (n=8)
Figure 4G	Peak $Na_V1.7$ current density	Unpaired t test	PA (10 μM) vs 2-BP (25 μM)<br>C1152A: p=0.0415	C1152A: PA (10 μM) (n=12)<br>2-BP (25 μM) (n=11)
Figure 4H	$V_{1/2}$ of activation	Unpaired t test	PA (10 μM) vs 2-BP (25 μM)<br>C1152A: p<0.0001	C1152A: PA (10 μM) (n=12)<br>2-BP (25 μM) (n=10)
Figure 4I	$V_{1/2}$ of inactivation	Unpaired t test	PA (10 μM) vs 2-BP (25 μM)<br>C1152A: p= 0.0886	C1152A: PA (10 μM) (n=10)<br>2-BP (25 μM) (n=11)

Figure 4K	Time constant (τ) of recovery from inactivation	Kruskal-Wallis test<br>p=0.0010	Na$_V$1.7-WT vs Na$_V$1.7-C1126A p=0.2311<br><br>Na$_V$1.7-WT vs Na$_V$1.7-C1152A p=0.0516	Na$_V$1.7-WT (n=20)<br>Na$_V$1.7-C1126A (n=14)<br>Na$_V$1.7-C1152A (n=18)
Figure 4M	Plateau ratio of slow inactivation	Kruskal-Wallis test<br>p=0.0948	Na$_V$1.7-WT vs Na$_V$1.7-C1126A p>0.9999<br><br>Na$_V$1.7-WT vs Na$_V$1.7-C1152A p=0.0978<br><br>Na$_V$1.7-C1126A vs Na$_V$1.7-C1152A p=0.5308	Na$_V$1.7-WT (n=15)<br>Na$_V$1.7-C1126A (n=14)<br>Na$_V$1.7-C1152A (n=16)
Figure 5C	*S*-palmitoylated Na$_V$1.7	One-way ANOVA<br>p=0.2125	Tukey's multiple comparisons Test<br>Na$_V$1.7-WT 0.1% DMSO vs Na$_V$1.7-WT PA (10 μM): p=0.9933<br>Na$_V$1.7-WT 0.1% DMSO vs Na$_V$1.7-WT 2-BP (25 μM): p=0.8428<br>Na$_V$1.7-WT 0.1% DMSO vs Na$_V$1.7-C1126A 0.1% DMSO: p=0.9925<br>Na$_V$1.7-WT 0.1% DMSO vs Na$_V$1.7-C1126A PA (10 μM): p=0.5883<br>Na$_V$1.7-WT 0.1% DMSO vs Na$_V$1.7-C1126A 2-BP (25 μM): p=0.9994<br>Na$_V$1.7-WT 0.1% DMSO vs Na$_V$1.7-C1152A 0.1% DMSO: p=0.9994<br>Na$_V$1.7-WT 0.1% DMSO vs Na$_V$1.7-C1152A PA (10 μM): p=0.9997<br>Na$_V$1.7-WT 0.1% DMSO vs Na$_V$1.7-C1152A 2-BP (25 μM): p>0.9999	Na$_V$1.7-C1126A 0.1% DMSO (n=3)<br><br>Na$_V$1.7-C1126A PA (10 μM) (n=4)<br><br>Na$_V$1.7-C1126A 2-BP (25 μM) (n=4)<br><br>Na$_V$1.7-C1152A 0.1% DMSO (n=4)<br><br>Na$_V$1.7-C1152A PA (10 μM) (n=4)<br><br>Na$_V$1.7-C1152A 2-BP (25 μM) (n=4)

			Na$_V$1.7-WT PA (10 µM) vs Na$_V$1.7-WT 2-BP (25 µM): p=0.3224<br>Na$_V$1.7-WT PA (10 µM) vs Na$_V$1.7-C1126A 0.1% DMSO: p>0.9999<br>Na$_V$1.7-WT PA (10 µM) vs Na$_V$1.7-C1126A PA (10 µM): p=0.9432<br>Na$_V$1.7-WT PA (10 µM) vs Na$_V$1.7-C1126A 2-BP (25 µM): p>0.9999<br>Na$_V$1.7-WT PA (10 µM) vs Na$_V$1.7-C1152A 0.1% DMSO: p>0.9999<br>Na$_V$1.7-WT PA (10 µM) vs Na$_V$1.7-C1152A PA (10 µM): p=0.9308<br>Na$_V$1.7-WT PA (10 µM) vs Na$_V$1.7-C1152A 2-BP (25 µM): p=0.9978<br>Na$_V$1.7-WT 2-BP (25 µM) vs Na$_V$1.7-C1126A 0.1% DMSO: p=0.5513<br>Na$_V$1.7-WT 2-BP (25 µM) vs Na$_V$1.7-C1126A PA (10 µM): p=0.0644<br>Na$_V$1.7-WT 2-BP (25 µM) vs Na$_V$1.7-C1126A 2-BP (25 µM): p=0.6479<br>Na$_V$1.7-WT 2-BP (25 µM) vs Na$_V$1.7-C1152A 0.1% DMSO: p=0.6538<br>Na$_V$1.7-WT 2-BP (25 µM) vs Na$_V$1.7-C1152A PA (10 µM): p=0.9990<br>Na$_V$1.7-WT 2-BP (25 µM) vs Na$_V$1.7-C1152A 2-	

			BP (25 µM): p=0.9506<br>Na$_V$1.7-C1126A 0.1% DMSO vs Na$_V$1.7-C1126A PA (10 µM): p=0.9969<br>Na$_V$1.7-C1126A 0.1% DMSO vs Na$_V$1.7-C1126A 2-BP (25 µM): p>0.9999<br>Na$_V$1.7-C1126A 0.1% DMSO vs Na$_V$1.7-C1152A 0.1% DMSO: p>0.9999<br>Na$_V$1.7-C1126A 0.1% DMSO vs Na$_V$1.7-C1152A PA (10 µM): p=0.9414<br>Na$_V$1.7-C1126A 0.1% DMSO vs Na$_V$1.7-C1152A 2-BP (25 µM): p=0.9959<br>Na$_V$1.7-C1126A PA (10 µM) vs Na$_V$1.7-C1126A 2-BP (25 µM): p=0.9622<br>Na$_V$1.7-C1126A PA (10 µM) vs Na$_V$1.7-C1152A 0.1% DMSO: p=0.9606<br>Na$_V$1.7-C1126A PA (10 µM) vs Na$_V$1.7-C1152A PA (10 µM): p=0.4388<br>Na$_V$1.7-C1126A PA (10 µM) vs Na$_V$1.7-C1152A 2-BP (25 µM): p=0.7360<br>Na$_V$1.7-C1126A 2-BP (25 µM) vs Na$_V$1.7-C1152A 0.1% DMSO: p>0.9999<br>Na$_V$1.7-C1126A 2-BP (25 µM) vs Na$_V$1.7-C1152A	

			PA (10 µM):<br>p=0.9803<br>Na$_V$1.7-C1126A 2-BP (25 µM) vs Na$_V$1.7-C1152A 2-BP (25 µM):<br>p=0.9997<br>Na$_V$1.7-C1152A 0.1% DMSO vs Na$_V$1.7-C1152A PA (10 µM):<br>p=0.9812<br>Na$_V$1.7-C1152A 0.1% DMSO vs Na$_V$1.7-C1152A 2-BP (25 µM):<br>p=0.9997<br>Na$_V$1.7-C1152A PA (10 µM) vs Na$_V$1.7-C1152A 2-BP (25 µM):<br>p>0.9999	
Figure 6C	Peak Na$_V$1.7 current density	One-way ANOVA<br>p=0.1266	Tukey's multiple comparisons Test<br>Na$_V$1.7-C1126A/C1152A 0.1% DMSO vs Na$_V$1.7-C1126A/C1152A PA (10 µM):<br>p=0.9558<br>Na$_V$1.7-C1126A/C1152A 0.1% DMSO vs Na$_V$1.7-C1126A/C1152A 2-BP (25 µM):<br>p= 0.2976<br>Na$_V$1.7-C1126A/C1152A PA (10 µM) vs Na$_V$1.7-C1126A/C1152A 2-BP (25 µM):<br>p= 0.1283	Na$_V$1.7-C1126A/C1152A 0.1% DMSO (n=22)<br><br>Na$_V$1.7-C1126A/C1152A PA (10 µM) (n=31)<br><br>Na$_V$1.7-C1126A/C1152A 2-BP (25 µM) (n=27)
Figure 6D	$V_{1/2}$ of activation	Unpaired t test	PA (10 µM) vs 2-BP (25 µM) C1126A/C1152A:<br>p=0.6088	C1126A/C1152A:<br>PA (10 µM) (n=29)<br>2-BP (25 µM) (n=27)
Figure 6D	$V_{1/2}$ of inactivation	Unpaired t test	PA (10 µM) vs 2-BP (25 µM) C1126A/C1152A:<br>p=0.2699	C1126A/C1152A:<br>PA (10 µM) (n=28)<br>2-BP (25 µM) (n=26)

Figure 6H	Plateau ratio of slow inactivation	Unpaired t test	$Na_V1.7$-WT vs $Na_V1.7$-C1126A/C1152A p=0.0995	$Na_V1.7$-WT (n=15) $Na_V1.7$-C1126A/C1152A (n=9)
Figure 7B	Normalized *S*-palmitoylated $Na_V1.7$	One-way ANOVA p=0.0028	$Na_V1.7$-WT 0.1% DMSO vs $Na_V1.7$-WT PA (10 μM): p=0.8860<br>$Na_V1.7$-WT 0.1% DMSO vs $Na_V1.7$-WT 2-BP (25 μM): p=0.4362<br>$Na_V1.7$-WT 0.1% DMSO vs $Na_V1.7$-C1126A/C1152A 0.1% DMSO: p=0.1209<br>$Na_V1.7$-WT 0.1% DMSO vs $Na_V1.7$-C1126A/C1152A PA (10 μM): p=0.1904<br>$Na_V1.7$-WT 0.1% DMSO vs $Na_V1.7$-C1126A/C1152A 2-BP (25 μM): p=0.2700<br>$Na_V1.7$-WT PA (10 μM) vs $Na_V1.7$-WT 2-BP (25 μM): p=0.0609<br>$Na_V1.7$-WT PA (10 μM) vs $Na_V1.7$-C1126A/C1152A 0.1% DMSO: p=0.0164<br>$Na_V1.7$-WT PA (10 μM) vs $Na_V1.7$-C1126A/C1152A PA (10 μM): p=0.0290<br>$Na_V1.7$-WT PA (10 μM) vs $Na_V1.7$-C1126A/C1152A 2-BP (25 μM): p=0.0464<br>$Na_V1.7$-WT 2-BP (25 μM) vs $Na_V1.7$-C1126A/C1152A 0.1% DMSO: p=0.8778<br>$Na_V1.7$-WT 2-BP (25 μM) vs $Na_V1.7$-	$Na_V1.7$-WT 0.1% DMSO (n=8)<br><br>$Na_V1.7$-WT PA (10 μM) (n=8)<br><br>$Na_V1.7$-WT 2-BP (25 μM) (n=8)<br><br>$Na_V1.7$-C1126A/C1152A 0.1% DMSO (n=4)<br><br>$Na_V1.7$-C1126A/C1152A PA (10 μM) (n=4)<br><br>$Na_V1.7$-C1126A/C1152A 2-BP (25 μM) (n=4)

			C1126A/C1152A PA (10 μM): p=0.9523<br>NaV1.7-WT 2-BP (25 μM) vs NaV1.7-C1126A/C1152A 2-BP (25 μM): p=0.9846<br>NaV1.7-C1126A/C1152A 0.1% DMSO vs NaV1.7-C1126A/C1152A PA (10 μM): p>0.9999<br>NaV1.7-C1126A/C1152A 0.1% DMSO vs NaV1.7-C1126A/C1152A 2-BP (25 μM): p=0.9989<br>NaV1.7-C1126A/C1152A PA (10 μM) vs NaV1.7-C1126A/C1152A 2-BP (25 μM): p>0.9999	
Figure 8B	Excitability-Evoked action potentials per step	Two-way ANOVA	Tukey's multiple comparisons Test<br>10 pA<br>0.1% DMSO vs PA (10 μM): p=0.6196<br>0.1% DMSO vs 2-BP (25 μM): p=0.0073<br>PA (10 μM) vs 2-BP (25 μM): p=0.0976<br><br>20 pA<br>0.1% DMSO vs PA (10 μM): p=0.8290<br>0.1% DMSO vs 2-BP (25 μM): p=0.0112<br>PA (10 μM) vs 2-BP (25 μM): p=0.1573<br><br>30 pA	0.1% DMSO (n=12)<br>PA (10 μM) (n=7)<br>2-BP (25 μM) (n=11)

			0.1% DMSO vs PA (10 μM): p=0.7828<br>0.1% DMSO vs 2-BP (25 μM): p=0.0074<br>PA (10 μM) vs 2-BP (25 μM): p=0.0963<br><br>40 pA<br>0.1% DMSO vs PA (10 μM): p=0.8019<br>0.1% DMSO vs 2-BP (25 μM): p=0.0048<br>PA (10 μM) vs 2-BP (25 μM): p=0.0665<br><br>50 pA<br>0.1% DMSO vs PA (10 μM): p=0.7746<br>0.1% DMSO vs 2-BP (25 μM): p=0.0077<br>PA (10 μM) vs 2-BP (25 μM): p=0.1245<br><br>60 pA<br>0.1% DMSO vs PA (10 μM): p=0.8177<br>0.1% DMSO vs 2-BP (25 μM): p=0.0114<br>PA (10 μM) vs 2-BP (25 μM): p=0.1328<br><br>70 pA<br>0.1% DMSO vs PA (10 μM): p=0.7079<br>0.1% DMSO vs 2-BP (25 μM): p=0.0041<br>PA (10 μM) vs 2-BP (25 μM): p=0.0810<br><br>80 pA	

			0.1% DMSO vs PA (10 µM): p=0.8100<br>0.1% DMSO vs 2-BP (25 µM): p=0.0107<br>PA (10 µM) vs 2-BP (25 µM): p=0.1079<br><br>90 pA<br>0.1% DMSO vs PA (10 µM): p=0.4085<br>0.1% DMSO vs 2-BP (25 µM): p=0.0062<br>PA (10 µM) vs 2-BP (25 µM): p=0.1540<br><br>100 pA<br>0.1% DMSO vs PA (10 µM): p=0.5934<br>0.1% DMSO vs 2-BP (25 µM): p=0.0051<br>PA (10 µM) vs 2-BP (25 µM): p=0.0806<br><br>110 pA<br>0.1% DMSO vs PA (10 µM): p=0.5248<br>0.1% DMSO vs 2-BP (25 µM): p=0.0024<br>PA (10 µM) vs 2-BP (25 µM): p=0.1040<br><br>120 pA<br>0.1% DMSO vs PA (10 µM): p=0.8548<br>0.1% DMSO vs 2-BP (25 µM): p=0.0203<br>PA (10 µM) vs 2-BP (25 µM): p=0.1292	
Figure 8C	Excitability-Resting	One-way ANOVA p=0.0297	Tukey's multiple comparisons Test	0.1% DMSO (n=12)<br>PA (10 µM) (n=7)

	membrane potential		0.1% DMSO vs PA (10 μM): p=0.0637<br>0.1% DMSO vs 2-BP (25 μM): p= 0.9059<br>PA (10 μM) vs 2-BP (25 μM): p=0.0312	2-BP (25 μM) (n=11)
Figure 8E	Excitability-Rheobase	One-way ANOVA p=0.0267	Tukey's multiple comparisons Test<br>0.1% DMSO vs PA (10 μM): p=0.9414<br>0.1% DMSO vs 2-BP (25 μM): p= 0.0282<br>PA (10 μM) vs 2-BP (25 μM): p=0.1224	0.1% DMSO (n=12)<br>PA (10 μM) (n=7)<br>2-BP (25 μM) (n=11)
Figure 9C	$V_{1/2}$ of activation	One-way ANOVA p<0.0001	Tukey's multiple comparisons Test<br>0.1% DMSO vs PA (10 μM): p=0.0875<br>0.1% DMSO vs 2-BP (25 μM): p<0.0001<br>PA (10 μM) vs 2-BP (25 μM): p=0.0033	0.1% DMSO (n=9)<br>PA (10 μM) (n=7)<br>2-BP (25 μM) (n=9)
Figure 9C	$V_{1/2}$ of inactivation	One-way ANOVA p=0.7501	Tukey's multiple comparisons Test<br>0.1% DMSO vs PA (10 μM): p=0.9859<br>0.1% DMSO vs 2-BP (25 μM): p= 0.8342<br>PA (10 μM) vs 2-BP (25 μM): p=0.7561	0.1% DMSO (n=8)<br>PA (10 μM) (n=7)<br>2-BP (25 μM) (n=8)
Figure 9F	Human Excitability-Resting membrane potential	Kruskal-Wallis test p=0.1435	Dunn's multiple comparisons Test<br>0.1% DMSO vs PA (10 μM): p=0.2376<br>0.1% DMSO vs 2-BP (25 μM): p>0.9999<br>PA (10 μM) vs 2-BP (25 μM):	0.1% DMSO (n=11)<br>PA (10 μM) (n=11)<br>2-BP (25 μM) (n=14)

			p=0.2741	
Figure 9G	Human Excitability-Rheobase	Kruskal-Wallis test p=0.1785	Dunn's multiple comparisons Test<br>0.1% DMSO vs PA (10 μM): p=0.7154<br>0.1% DMSO vs 2-BP (25 μM): p>0.9999<br>PA (10 μM) vs 2-BP (25 μM): p=0.1970	0.1% DMSO (n=11)<br>PA (10 μM) (n=11)<br>2-BP (25 μM) (n=14)
Figure S1A	Peak $Na_V1.7$ current density	Multiple unpaired t test	Multiple unpaired t test; PA (10 μM) vs 2-BP (25 μM)<br>WT: p=0.1889<br>C709A: p=0.0113<br>C1163A: p=0.0004<br>C1164A: p=0.0008<br>C1183A: p=0.0162<br>C1257A: p=0.0209<br>C1798A: p=0.0054<br>C1836A: p=0.0043	WT: PA (10 μM) (n=22)<br>2-BP (25 μM) (n=24)<br><br>C709A: PA (10 μM) (n=12)<br>2-BP (25 μM) (n=13)<br><br>C1163A: PA (10 μM) (n=13)<br>2-BP (25 μM) (n=13)<br><br>C1164A: PA (10 μM) (n=11)<br>2-BP (25 μM) (n=13)<br><br>C1183A: PA (10 μM) (n=13)<br>2-BP (25 μM) (n=13)<br><br>C1257A: PA (10 μM) (n=14)<br>2-BP (25 μM) (n=14)<br><br>C1798A: PA (10 μM) (n=14)<br>2-BP (25 μM) (n=10)<br><br>C1836A: PA (10 μM) (n=10)2-BP (25 μM) (n=11)
Figure S1B	$V_{1/2}$ of inactivation	Multiple unpaired t test	Multiple unpaired t test; PA (10 μM) vs 2-BP (25 μM)<br>WT: p<0.0001<br>C709A: p=0.0011	WT: PA (10 μM) (n=21) 2-BP (25 μM) (n=23)

			C1163A: p=0.0001<br>C1164A: p<0.0001<br>C1183A: p=0.0005<br>C1257A: p=0.0094<br>C1798A: p=0.0028<br>C1836A: p=0.0008	C709A: PA (10 µM) (n=14)<br>2-BP (25 µM) (n=13)<br><br>C1163A: PA (10 µM) (n=11)<br>2-BP (25 µM) (n=12)<br><br>C1164A: PA (10 µM) (n=12)<br>2-BP (25 µM) (n=13)<br><br>C1183A: PA (10 µM) (n=14)<br>2-BP (25 µM) (n=14)<br><br>C1257A: PA (10 µM) (n=14)<br>2-BP (25 µM) (n=14)<br><br>C1798A: PA (10 µM) (n=14)<br>2-BP (25 µM) (n=13)<br><br>C1836A: PA (10 µM) (n=10) 2-BP (25 µM) (n=10)
Figure S2D	$V_{1/2}$ of inactivation	One-way ANOVA<br>p=0.08101	Tukey's multiple comparisons Test<br>0.1% DMSO vs PA (10 µM): p=0.9660<br>0.1% DMSO vs 2-BP (25 µM): p= 0.9858<br>PA (10 µM) vs 2-BP (25 µM): p=0.9154	0.1% DMSO (n=9)<br>PA (10 µM) (n=7)<br>2-BP (25 µM) (n=9)
